# Global range expansion history of pepper (*Capsicum* spp.) revealed by over 10,000 genebank accessions

**DOI:** 10.1073/pnas.2104315118

**Published:** 2021-08-16

**Authors:** Pasquale Tripodi, Mark Timothy Rabanus-Wallace, Lorenzo Barchi, Sandip Kale, Salvatore Esposito, Alberto Acquadro, Roland Schafleitner, Maarten van Zonneveld, Jaime Prohens, Maria José Diez, Andreas Börner, Jérémy Salinier, Bernard Caromel, Arnaud Bovy, Filiz Boyaci, Gancho Pasev, Ronny Brandt, Axel Himmelbach, Ezio Portis, Richard Finkers, Sergio Lanteri, Ilan Paran, Véronique Lefebvre, Giovanni Giuliano, Nils Stein

**Affiliations:** ^a^Research Centre for Vegetable and Ornamental Crops, Council for Agricultural Research and Economics (CREA), 84098 Pontecagnano Faiano, Italy;; ^b^Genome Diversity, Department Genebank, Leibniz Institute of Plant Genetics and Crop Plant Research (IPK), 06466 Seeland, Germany;; ^c^Department of Agricultural, Forest and Food Sciences (DISAFA), Plant Genetics, University of Torino, 10095 Grugliasco, Italy;; ^d^Genetic Resources and Seed Unit, World Vegetable Centre, Shanhua 74151, Taiwan;; ^e^Instituto de Conservación y Mejora de la Agrodiversidad Valenciana, Universitat Politècnica de València (UPV), 46022 Valencia, Spain;; ^f^Unité de Génétique et Amélioration des Fruits et Légumes (GAFL), L’Institut National de Recherche pour l’Agriculture, l’Alimentation et l’Environnement (INRAE), F-84140 Montfavet, France;; ^g^Plant Breeding, Wageningen University & Research, 6700 AJ Wageningen, The Netherlands;; ^h^Bati Akdeniz Agricultural Research Institute, 07100 Antalya, Turkey;; ^i^Maritsa Vegetable Crops Research Institute, 4003 Plovdiv, Bulgaria;; ^j^Institute of Plant Sciences, Agricultural Research Organization, The Volcani Center, Bet Dagan 50250, Israel;; ^k^Casaccia Research Centre, Italian National Agency for New Technologies, Energy, and Sustainable Economic Development, 00123 Rome, Italy;; ^l^Center for Integrated Breeding Research, Georg-August-University Göttingen, 37075 Göttingen, Germany

**Keywords:** genebank, pepper, population genomics, routes of diversification, GWAS

## Abstract

Genebanks collect and preserve vast collections of plants and detailed passport information, with the aim of preserving genetic diversity for conservation and breeding. Genetic characterization of such collections has the potential to elucidate the genetic histories of important crops, use marker–trait associations to identify loci controlling traits of interest, search for loci undergoing selection, and contribute to genebank management by identifying taxonomic misassignments and duplicates. We conducted a genomic scan with genotyping by sequencing (GBS) derived single nucleotide polymorphisms (SNPs) of 10,038 pepper (*Capsicum* spp.) accessions from worldwide genebanks and investigated the recent history of this iconic staple. Genomic data detected up to 1,618 duplicate accessions within and between genebanks and showed that taxonomic ambiguity and misclassification often involve interspecific hybrids that are difficult to classify morphologically. We deeply interrogated the genetic diversity of the commonly consumed *Capsicum annuum* to investigate its history, finding that the kinds of peppers collected in broad regions across the globe overlap considerably. The method *ReMIXTURE—*using genetic data to quantify the similarity between the complement of peppers from a focal region and those from other regions—was developed to supplement traditional population genetic analyses. The results reflect a vision of pepper as a highly desirable and tradable cultural commodity, spreading rapidly throughout the globe along major maritime and terrestrial trade routes. Marker associations and possible selective sweeps affecting traits such as pungency were observed, and these traits were shown to be distributed nonuniformly across the globe, suggesting that human preferences exerted a primary influence over domesticated pepper genetic structure.

## Significance

This study provides a deep population genomic analysis of 10,000 *Capsicum* accessions held in genebanks and representing a frame of the global diversity of the genus. By combining single nucleotide polymorphisms (SNPs) based data and passport information, we investigated the genomic diversity and population structure of wild and domesticated peppers, tracing back to routes of evolution and providing a model of *Capsicum annuum* distribution, which reflects human trade and historical/cultural influences. Our results highlight west–east routes of expansion, shedding light on the links between South and Mesoamerica, Africa, and East/South Asia, the latter two constituting important diversification centers of pepper diversity. Finally, we outline a roadmap for genebank management and future direction for better exploitation of germplasm resources.

During the early 20th century, awareness of the increasing loss of genetic diversity in crops (1) first prompted the establishment of genebanks. The Food and Agriculture Organization (FAO) estimated an overall 7.4 million germplasm accessions to be presently maintained ex situ in over 1,700 genebanks worldwide (2). This remarkable volume of preserved genetic material represents an invaluable resource for facing challenges due to climate change and increasing pressure on global food production pathways (3). The same conserved crop diversity—product of the evolutionary history of the species—can also be interrogated to dissect the primary historical forces leading to the establishment of crops as human culturally/agriculturally significant items. Genebanks generally aim to sample a representative range of the diversity grown or found in a given region, and the collection location information is conveniently stored as passport data. Thus, the genetic relationships among regional complements of genebank-preserved agricultural specimens can be juxtaposed against the history of human or commercial relationships, migration, invasion, etc. among those regions. Unfortunately, the exploitation of plant genetic resources (PGRs) for such purposes may be compromised by a lack of metadata, including information on geographical origin and phenotype. Genomic approaches such as large-scale genotyping have shown great potential to address such problems in genebank management, in particular the merging of duplicate samples with distinct information and the correction of taxonomic misassignments (4, 5).

The *Capsicum* gene pool comprises domesticated and wild species with highly variable morphology and flavor and characterized by variable levels of pungency due to the presence of capsaicinoids that made peppers a staple cultural component in cuisines across the globe (6). This broad diversity is due to evolutionary and domestication processes that occurred in the centers of origin located in Mesoamerica and the Andes and to subsequent selective pressures associated with cultivation in tropical and temperate environments across all major equatorial continents (7), leading to the domestication of five *Capsicum* species (*Capsicum annuum* L., *Capsicum frutescens* L., *Capsicum chinense* Jacq., *Capsicum baccatum* L., and *Capsicum pubescens* Ruiz and Pav.), of which *C. annuum* is globally the most cultivated. Historical records suggest that pepper was brought by Columbus from the New World to Europe and was thenceforth traded along most major maritime and overland trade routes. However, records are scattered, and controversies remain regarding the possibility of East/Southeast-Asian connections to the Americas independent of—and perhaps earlier than—the establishment of transatlantic trade routes by Europeans during the 16th century. The historical and archaeological records have not yet been synthesized with genetic data at a global scale, although the germplasm suitable for such a study is aptly represented among genebank collections. The full landscape of genomic diversity available in *Capsicum* PGRs has been only partially exploited, being applied to limited subsets (8–10), and/or at a low genetic markers scale (11, 12).

Here, we report the sequencing-based genotyping of 10,038 accessions from 14 *Capsicum* species and subspecies stored in major international genebanks and research institutions. This collection effectively samples the global genetic diversity of pepper spanning the intertropical and temperate regions of the globe. From the genomic inferences on the massive scale of data, we examined the ex situ population structure, providing insight for better management and linking it to the expansion of *C. annuum*, with a particular focus on the way human trade is reflected in the genetic diversity and differentiation of varieties sampled from different regions. Then, we exploited archives of phenotypic data for scanning loci selected during the evolution of the crop.

## Results and Discussion

### Detection of Redundancy and Species Misassignment in Global Ex Situ Pepper Genebanks.

The sharing and inconsistent documentation of germplasm often results in duplicates within and between genebanks that can be hard or impossible to identify and could affect population genomic analyses (13). To identify tentatively duplicated samples, based on the genotype matrix (26,566 single nucleotide polymorphisms [SNPs]) derived from 10,038 pepper accessions (*SI Appendix*, Table S1 and Dataset S1), we estimated identity-by-state (IBS) proportions considering all pairwise combinations of accessions. We added a set of control replicates to the dataset (in this case, 224 replicate samples of *C. annuum* cv. “CM334”) to discover what range of IBS proportions would be assigned to samples known to be identical replicates. A strongly bimodal distribution of IBS with one peak close to IBS = 0 was observed, with this peak containing all the “CM334” versus “CM334” comparisons. This allowed us to impose a cutoff value of IBS < 0.0001 for considering two accessions as potential duplicates (*SI Appendix*, Fig. S1*A*). A total of 2,353 noncontrol accessions clustered in 735 groups (*SI Appendix*, Fig. S1*B*), thus revealing that 1,618 accessions were genetic duplicates of other accessions, with the cardinality ranging from 2 (526 groups) to 246 accessions (1 group). The fraction of duplicated accessions differed among the genebanks that were sourced for the analyzed germplasm, as well as between taxa. On average, ∼75% of germplasm was found to be unique within genebanks, with a fraction of unique accessions ranging from 63.5% at the Leibniz Institute of Plant Genetics and Crop Plant Research (IPK) to 80.4% at the Universitat Politècnica de València (UPV) (*SI Appendix*, Fig. S2*A*). A consistent proportion of shared samples was found between World Vegetable Centre (WorldVeg) and IPK (23.1%), whereas only a few accessions (8) were in common among all the five main genebanks (*SI Appendix*, Fig. S2*B*). Overall, duplication occurred mostly within the Annuum clade (*C. annuum* ∼78%; *C. frutescens* ∼10%; *C. chinense* ∼5%) (Dataset S2). These differences presumably reflect variation in both the deduplication practices of genebanks and difficulty with which novel entry accessions can be checked against the current collection. This significant level of duplication should motivate the development of genetic prescreening protocols to be used in genebanks for documenting the potential duplicate samples upon first acquisition.

To correct and impute species assignments (13), hierarchical clusters were imputed at a stringent Z-score threshold (Dataset S3). Members of clusters containing multiple species designations were assigned to the majority designation, if the majority assignment made up more than 80% of the cluster, after visual inspection of the principal component analysis (PCA) plots to assure the cluster did not fall within a region of broad overlap between multiple species. In total, 53 clusters included accessions from more than one species or undefined accessions. The largest part of species misassignment occurred within the Annuum clade and between the three *C. baccatum* groups, including the two botanical varieties and an undefined group. Rather than to misassignment, these outliers could be attributed to allele introgressions due to spontaneous crosses. While the levels of mis- and nonassignment of genebank taxa are expected to vary across taxonomic groups and to become more inaccurate at lower taxonomic levels, these results clearly demonstrate that genomics-based methods, integrated with passport information, should be considered the gold standard to correct any inaccuracy in species/subspecies assignment and duplicate detection.

**Fig. 1.**
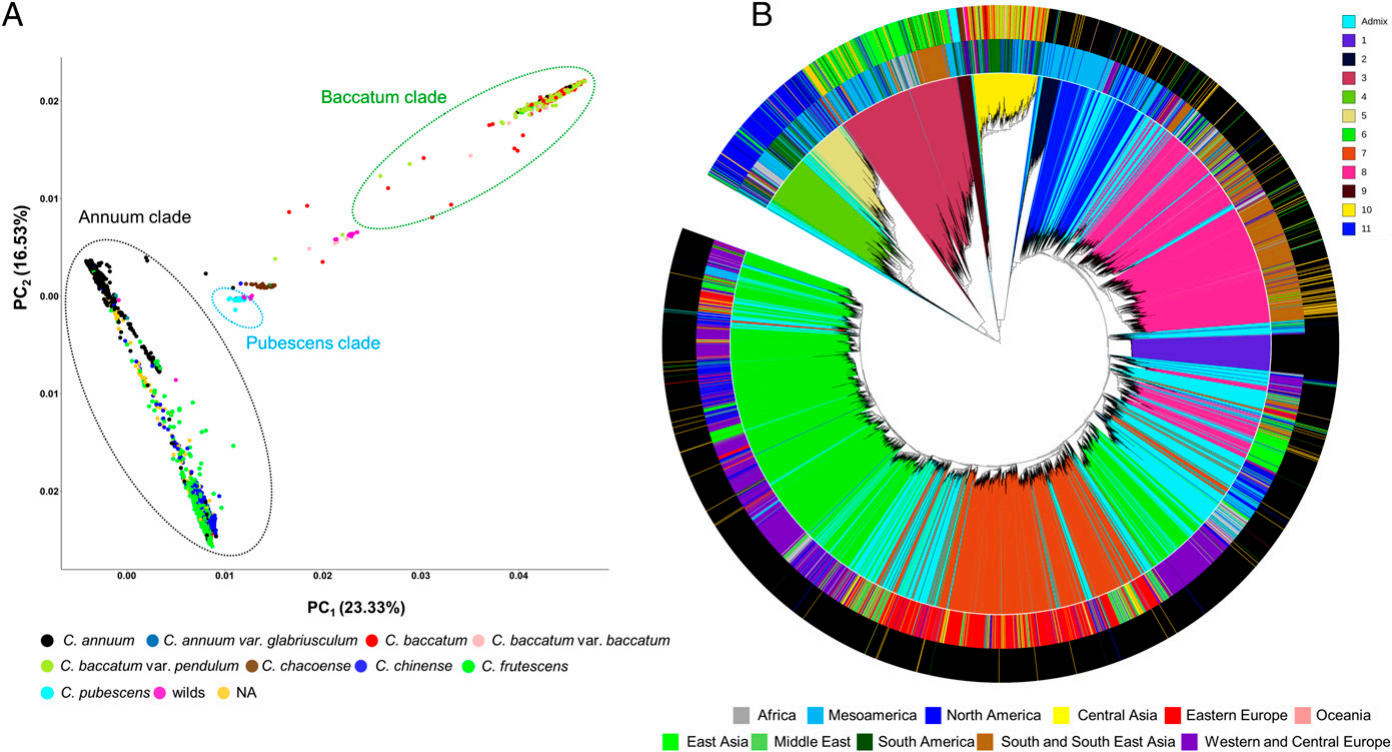
Pepper diversity in worldwide genebank holdings. (*A*) PCA plots in the first two components, showing genetic diversity among the full (undeduplicated) data set of 10,262 *Capsicum* accessions (10,038 samples and 234 control accessions). Samples are colored according to species (*Lower Left*). The first three components separate species according to taxonomy classification. (*B*) Combined unrooted phylogenetic three with ADMIXTURE analysis of the full (undeduplicated) dataset of *Capsicum* genotypes, with K = 11. The colors in the internal circle corresponds to clusters K1 to K11 (*Upper Right*), the intermediate circle corresponds to geographical origin (*Lower Right*) and external circle correspond to species (*Lower Left*). The black color in the intermediate cycle represents the experimental control accession *C. annuum* cv. “CM334.” The analysis confirms grouping accord to species showing more than a cluster for *C. chinense* (K4 and K5) and for *C. annuum* (K1, K2, K6, K7, K8, and K11).

Species assignments in the genus *Capsicum* generally reflect their membership of true genetic demes (Fig. 1*A*). Fitting a tree-based model to the data thus results in clade divisions closely corresponding to species assignments (Fig. 1*B*) (14, 15). Both *F*_*st*_ estimates (*SI Appendix*, Table S2) and PCA plot representations (Fig. 1*A* and *SI Appendix*, Fig. S3) were coherent with the main established complexes (15, 16). The *C. annuum* species (which is the most widely consumed and includes e.g., the popular Jalapeno, Cayenne, and Bell peppers) holds the largest diversity, alongside being best represented in the collection (and is hence used as the basis of demographic analyses discussed in *Pepper Range Expansion and Trading History Reflected in C. annuum Demography*). *C. baccatum* (a species with distinctive yellow-green spots in the corolla including several “Aji” cultivars and peculiar shape types like the popular “Bishop Crown”) is broadly separated from all other species groups. A significantly smaller degree of separation exists between *C. chinense* and *C. frutescens* accessions. *C. chinense* and *C. frutescens* are known for including the most pungent accessions, (e.g., Trinidad, Bhut Jolokia, and Habanero types; *C. chinense*), and the small-fruited Tabasco (*C. frutescens*) types giving the eponymous name to the sauce.

A cluster of taxonomically unclassified samples appears to connect to *C. annuum*, *C. chinense*, and *C. frutescens*, suggesting that difficult-to-classify individuals are often due to the frequent interbreeding events between these three species belonging to the Annuum clade (15), resulting in intermediate phenotypic traits (16)—which is reflected at the genetic level. While interspecies crossing may occur in the wild, accidental crossings during multiplication in genebanks might also contribute. In fact, we observed distinctly elevated heterozygosities, many in the range of 5 to 10%, within genetically intermediate samples, such as those falling in the Annuum clade (*SI Appendix*, Fig. S4 and Table S3), which are mostly sourced from the World Vegetable Center. While the frequency of accidental hybrids produced in a given genebank will vary with factors such as the multiplication techniques and the crossability between species, these higher heterozygosity levels do suggest the distinct possibility of recent, accidental crossings occurring in genebanks, leading to the creation of intermediate forms. However, many of them were marked as unclassified when they were collected, possibly resulting from natural or ancestral hybridization, and then maintaining a high rate of heterozygosity despite multiplication by selfing.

Broad overlap among *C. baccatum* accessions suggests a certain level of subspecies misassignment occurring within different repositories. *C. pubescens* and *C. chacoense*—both consumed to a small degree by humans—form distinct groups, and the data suggest that some wild individuals could arguably be collapsed into *C. pubescens*.

### Global Genomic Analysis of *Capsicum* Gene Pools Suggests Models of Domestication.

**Fig. 2.**
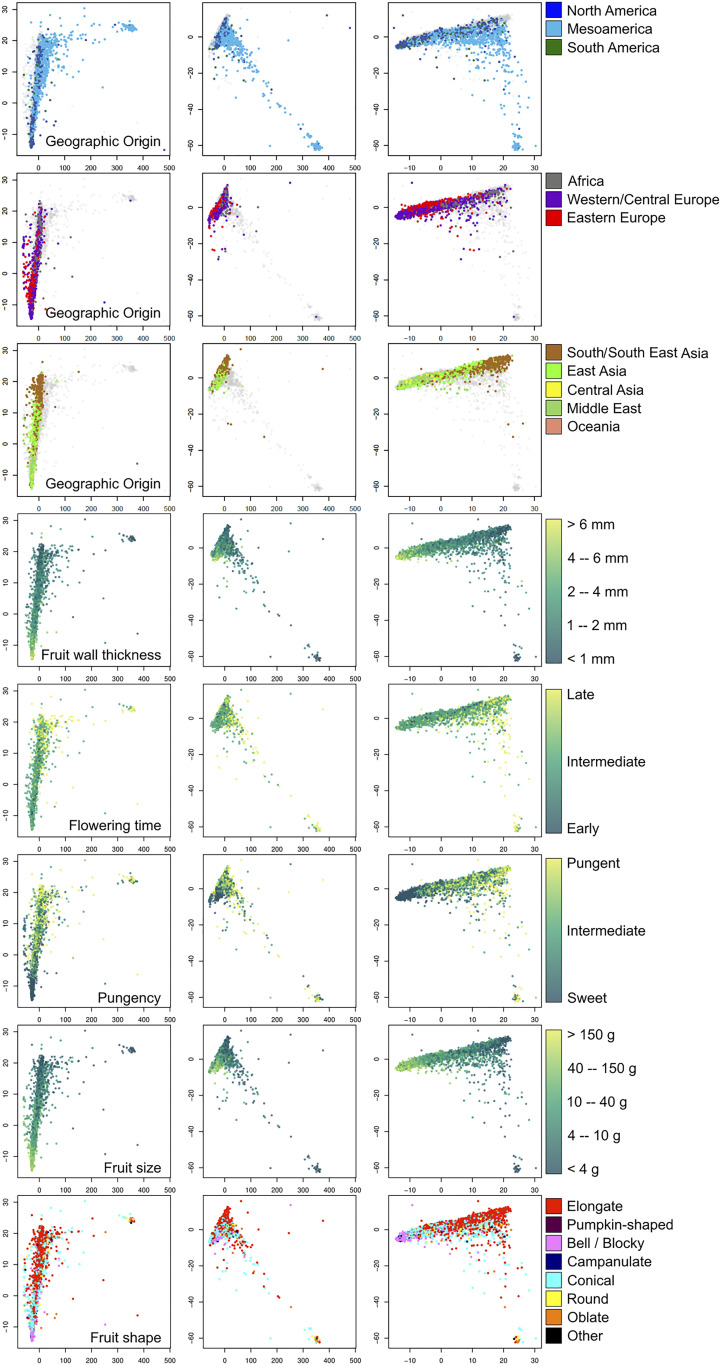
PCA plots showing the genetic diversity of *C. annuum* and its relation to geographical and phenotypic features (see labels *Inset* and keys *Right* of each row; region color code as in Fig. 1). Combinations of the first three PCs are shown left to right in each row (X/Y: PC1/PC2; PC1/PC3; PC2/PC3). PCs 1 to 3 explain 38.9, 25.7, and 12% of the total variation in the data, respectively. Fruit size was estimated according to weight ranges (*SI Appendix*, Table S9).

The ADMIXTURE algorithm models individuals as the products of variable levels of admixture between a collection of K genetic source groups. The results of running ADMIXTURE on the whole collection suggest that this model has limited appropriateness for pepper at the intraspecific scale (Fig. 1*B*). Indeed, while species were each typically assigned to a single dominant source group, the uniquely large amount of genetic diversity concentrated within *C. annuum* caused its members to be modeled as highly admixed combinations of five source populations (*SI Appendix*, Table S4). Application of the same method to the 7,848 *C. annuum* accessions alone suggested the species could be represented as combinations of nine highly admixed K clusters, roughly reflecting geographical divergences (*SI Appendix*, Fig. S5 *A*–*D* and Table S5). Evolutionary distances between clusters suggest the possible presence of more than one center of diversification (*SI Appendix*, Fig. S6). Using PCA plots to represent *C. annuum* population structure (Fig. 2) clearly reveals that region-wise geography does somewhat explain *C. annuum* genetic diversity, but the overlap between the peppers collected in different regions is far more striking than their separation (*SI Appendix*, Table S6). In general terms, European samples dominate one end of a cline, the other end of which is dominated by Asian samples, while samples collected across the Americas are fairly widely distributed along the cline. A second deme is populated mostly by Mesoamerican samples, and (rare) samples intermediate between the small deme and the main cline are found in most regions. These observations are more consistent with a high degree of founding diversity coupled with an intricately reticulate population history, suggesting a model of pepper domestication in which a wide range of representatives of a significant natural diversity pool originating in Mesoamerica (*SI Appendix*, Fig. S7) were widely domesticated and frequently transported and intercrossed. We expand on this concept with further analysis described in *Pepper Range Expansion and Trading History Reflected in C. annuum Demography* and *Conclusion*. Estimations of Tajima’s D show a consistent excess of rare alleles in *C. annuum* compared with other species (*SI Appendix*, Table S2) and also elevated *F*_*st*_ in most pairwise interspecific comparisons—both are compatible with a relatively stronger domestication bottleneck than other species, followed by population expansion and rapid dispersal to and between human cultures.

### Pepper Range Expansion and Trading History Reflected in *C. annuum* Demography.

Aiming to further leverage *C. annuum* diversity as a proxy for the human forces that have shaped it, we examined morphological and genetic similarities and differences between the *C. annuum* peppers found in global regions. To dissect this complexity further, we sought to visualize the data so to reflect interregion sharing of peppers. The method ReMIXTURE (”Regional Mixture”) was developed to establish the so-called relative genotypic overlaps (RGOs) between a selection of regions, to which *C. annuum* accessions were assigned based on genebank passport data (*SI Appendix*, Fig. S8).

**Fig. 3.**
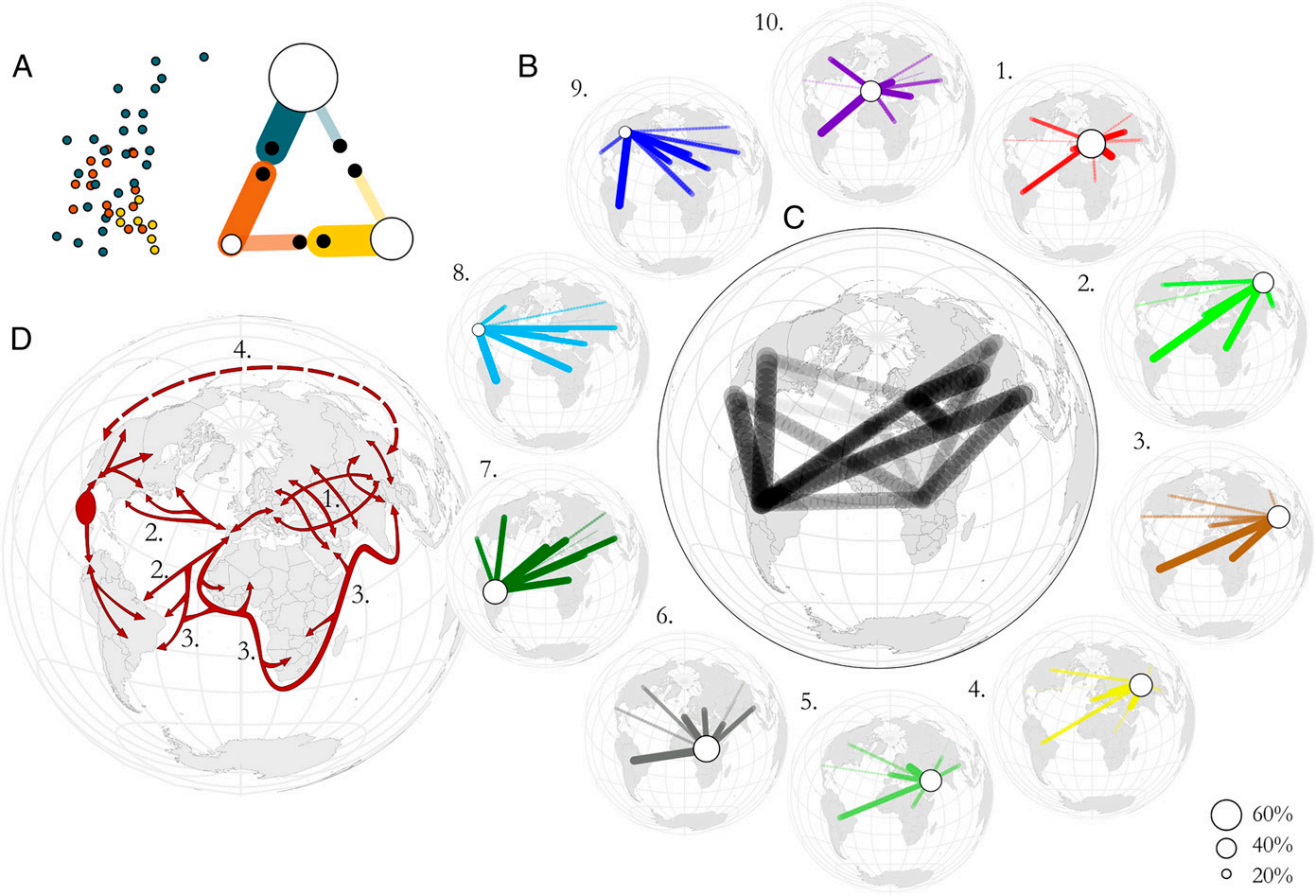
Region-wise pepper complement similarity using the ReMIXTURE method of calculating RGOs from each focal region. (*A*) Conceptual demonstration of the ReMIXTURE principle, with the “distances” between individuals of three hypothetical groups, shown as a two-dimensional scatterplot on *Left*, and hypothetical resulting ReMIXTURE output cartooned on *Right*. Thicker, more-opaque lines represent higher RGO with the focal region. The sizes of the circles at the center of each region represent the self RGO (“unique portion”) of the focal region. (*B*) (Outer ring) RGOs among 10 global regions (1 = Eastern Europe, 2 = East Asia, 3 = South and Southeast Asia, 4 = Central Asia, 5 = Middle East, 6 = Africa, 7 = South America, 8 = Mesoamerica, 9 = North America, and 10 = Central Europe. RGO according to color code used in Fig. 1*B*) (larger images in *SI Appendix*, Figs. S8–S12). The interregion RGO values range from 0.0009 to 25.9%. (*C*) Similarities between all regions, as in subfigure *B*, juxtaposed. The top three RGOs from each focal region are included. (*D*) Interpretation of major regional pepper-sharing vectors based on integration of the data. Numerals are referred to in *Pepper Range Expansion and Trading History Reflected in C. annuum Demography*.

The ReMIXTURE’s RGO_(region__A__→__region__B)_ measure provides an intuitive indication of the probability that a pepper accession chosen randomly from among the complement of pepper accessions in a focal region “A” will be genetically closer to a randomly chosen pepper accession in a target region “B” than it will belong to a randomly chosen pepper accession in any other region (Fig. 3*A*). Or, stated in another way, a focal region’s RGO profile expresses how one might approximate that region’s peppers by drawing a selection of peppers from other regions. Self-overlap (i.e., RGO_(region__A__→__region__A)_) can be understood as a proxy for uniqueness—the degree to which region A’s peppers cannot be well approximated by peppers from other regions. The RGOs—juxtaposed against the prevalence of certain phenotypes in certain regions—reflect several dominant themes that shaped the modern distribution of peppers. All in all, RGO tends to be higher between regions within the same continent, testifying to the influence of overland, riverine, and coastal trade (*SI Appendix*, Figs. S8–S12).

**Fig. 4.**
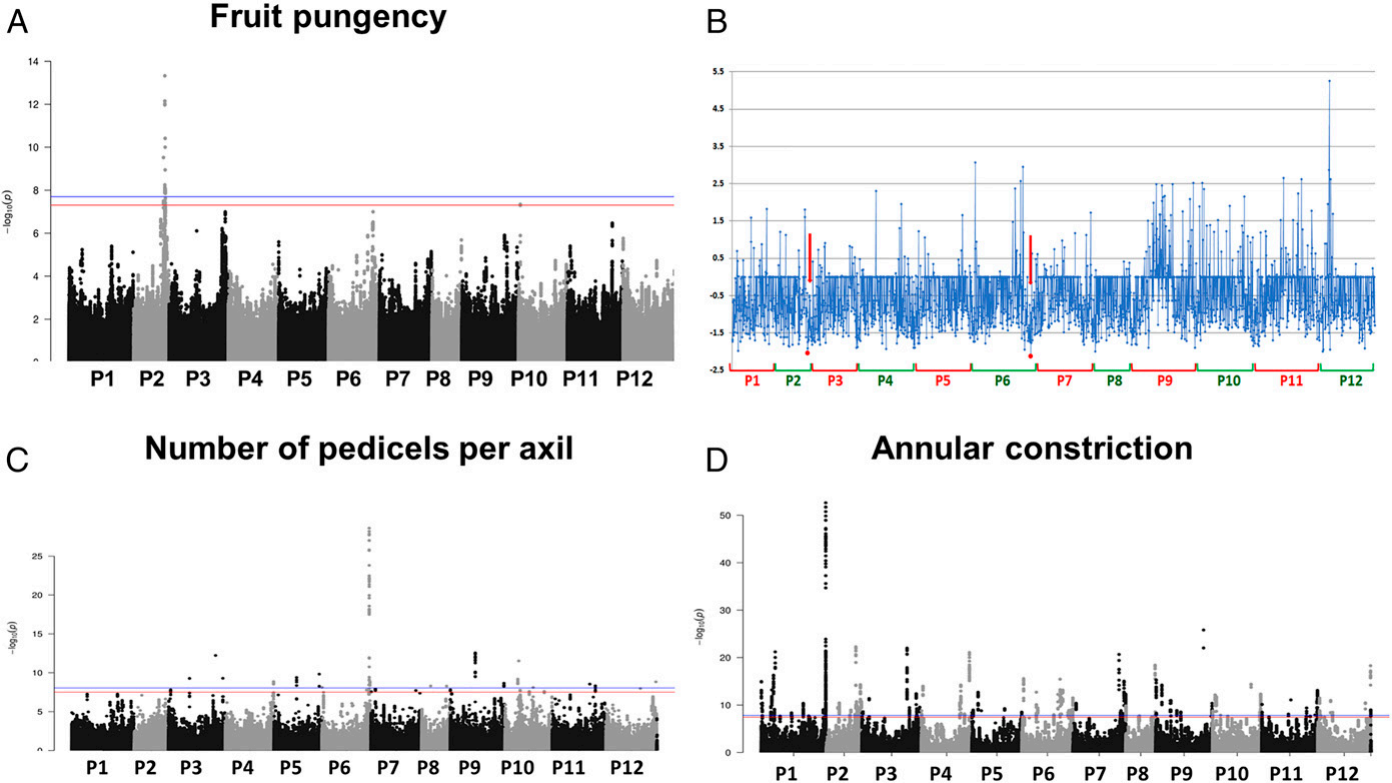
Genomic scans for selection and marker–trait associations in *C. annuum* across 12 pepper chromosomes (*Left* to *Right*). Manhattan plots showing GWAS associations for three traits (labels), of which one confirms previous studies (*A*) and two are associations for traits not yet fully explored (*C* and *D*). The blue line represents the genome-wide significance threshold (1.60 × 10^−8^), and the red line represents the suggestive association threshold (4.00 × 10^−8^). (*B*) Tajima’s D calculated in 1-Mb nonoverlapping bins, with putative historical selective sweeps resulting in locally depressed values identified by red arrows.

Overlapping between Western and Eastern pepper complements grown in Eurasia (Fig. 3 *B*, *1, 4, 10* and *C*) suggest routes such as the Silk Road certainly communicated pepper preferences along a latitudinal Eurasian axis, possibly facilitated by common day lengths and seasonalities, allowing pepper-friendly biomes to span the route (17). The mutually high RGOs connecting the Middle East to Central and Eastern Europe (Fig. 3 *B*, *1, 5, 10* and *C*) also suggest the influence of a longitudinal trade axis following Ottoman trade routes extending northwards from modern Turkey (18) (Fig. 3 *D*, *1*). Transcontinental overlaps between American and Eurasian pepper groups are more complex to interpret. *C. annuum* is thought to have been originated in Mexico (19, 20) after a clockwise expansion through South America and toward Central America (15). A multiregional model for subsequent domestication centered in Mexico has been suggested by Kraft et al. (21). Our data clearly confirm that South and Mesoamerican peppers span almost the entire main *C. annuum* diversity cline (Fig. 2) and contain considerable unique diversity reflected in high self RGO (*SI Appendix*, Fig. S8). Accordingly, pepper complements from regions in the eastern half of Eurasia have significant overlap with the South/Mesoamerican complement, most likely as a result of transatlantic trade during the 16th century (Fig. 3 *B*, *7, 8* and *D*, *2*): The peppers grown in these East Eurasian regions were primarily sourced—directly or indirectly—from the South/Mesoamerican diversity pool (Fig. 3*C*). The routes by which this pool came to the Old World are multiple. North America, perhaps owing to a historical cultural passion for diverse peppers, possesses an extremely diversified representation of the peppers of the world and likely served as an important vector for transatlantic pepper trade. Spanish trade routes connecting the Americas to Iberia no doubt played a key role in establishing these overlaps.

South America, while likely the origin of the wild peppers from which domesticated species arose, appears to have almost the entire domesticated *C. annuum* diversity presented in other regions. This is consistent with an initial domestication bottleneck occurring in Mesoamerica followed by the gradual accumulation of novel diversity and interbreeding following human-mediated dispersal from South America, as reflected by its high self RGO (*SI Appendix*, Fig. S8)

A particularly interesting signal involves the role of Africa in the human history of pepper. Africa forms a natural link between the Americas and the Eurasian pepper complements, likely owing in part to the triangular transatlantic slave trade and to Portuguese maritime trade routes around the Cape to connect with Arabic coastal trade along the southern margins of Eurasia (Fig. 3 *D*, *3*). This series of connections explains the African peppers’ overlap with those of both South America (to which Portuguese traders enjoyed privileged access following the 1494 Treaty of Tordesillas) and East/Central Asia (Fig. 3 *B*, *2, 3, 6*). The large proportion of the African complement that is unique may owe to gene flow as a consequence of germplasm exchange with the past trades (e.g., missionary settlements, colonial invasion, and slave trading) (22) combined with limited breeding activities that preserved the occurring ancient varieties.

Alongside Mesoamerica and Africa, self RGO values also confirm both Eastern Europe and East Asia as important stores of unique *C. annuum* diversity, the former a possible consequence of sweet and blocky peppers popular in European cuisines, resulting in additional centers of diversification (*SI Appendix*, Fig. S8 and Fig. 3 *B*, *1–3*). The fact that South American diversity can be best represented as a combination of East/South/Southeast-Asian and of African pepper diversity (Fig. 3*C* ) appears to support a connection between these three areas. Two main post-Columbian trade routes may be invoked to support this connection: the Portuguese empire trade route, joining the coastal colonies in Brazil, Africa, India, and China since the early 16th century (23) and the “silver route,” trading silver from the Spanish colonies of Perù and Mexico to China in the 17th century (24). A third, pre-Columbian and trans-Pacific route could be also invoked. This route could be the same through which sweet potato (*Ipomoea batatas*) was introduced from the Americas to Oceania and then to East Asia in historic times (25) and through which the bottle gourd (*Lagenaria siceraria*) followed the opposite route, being introduced from Africa to the Americas through Asia as early as 10,000 y ago (26).

The high North→South America and Meso→South America RGOs suggest a primary route of expansion through Americas (Fig. 3 *B**, 7*–*9* and *SI Appendix*, Fig. S8). In addition, the high South America→East/South/Southeast Asia RGO (Fig. 3*C* and *SI Appendix*, Fig. S8) favors the more modern, post-Columbian routes, particularly the Portuguese, highlighting South America as a possible gate of distribution. This would explain the lack of a high similarly Mesoamerica→Europe RGO, as the Portuguese did not have any great interest in introducing pungent peppers to Europe, thus creating a cheaper alternative to the highly valued black pepper (*Piper nigrum*), which they imported from the Far East (27). In contrast, they did have an incentive to introduce pungent peppers in the Far East as a cheaper surrogate for black pepper, thus favoring the import of the former to Europe. The East/South/Southeast-Asian pepper complement appears to be the result of access to many trading partners, coupled with a cultural impetus to accumulate pepper diversity 1) extremely broadly and 2) favoring, in particular, some common South and Mesomerican types. This “gatherers of diversity” scenario is analogous to the case of North America but would obviously have required much more extensive and intricate trade pathways. Under this interpretation, the observed South/Mesoamerica→East/South/Southeast Asia RGO must be interpreted as a consequence of preferential accumulation by East Asians of a significant representative portion of the kinds of peppers grown in Latin America, with these overlapping peppers being specifically less commonly accumulated in Western regions. This would cause a unilaterally inflated South America→East/South/Southeast Asia RGO, as is observed. We suggest this curious shared pocket of diversity is probably caused by a common culinary preference for (in general) small, red, hot peppers (Fig. 2). Why is this explanation preferable to a direct Sino-American link, followed by a degree of East-Asian isolation? We argue that 1) the volume of trade needed to homogenize two regions’ pepper complements would have left a greater body of uncontested historical evidence, 2) if such trade had dominated, it would have resulted in mutually (as opposed to unilaterally) elevated RGOs, and 3) it would not explain the elevated Asia→Africa RGO (Fig. 3 *B*, *2, 3*) and various elevated Europe→Asia/Asia→Europe RGOs (Fig. 3 *B*, *1, 4, 10*). All these arguments collectively provided a perfectly parsimonious pathway for the regular transfer of peppers from America to Asia via the Atlantic route (Fig. 3 *D*, *1*–*3*). The evidence does not rule out a Sino-American trade connection, but it does not require this scenario to explain East/South/Southeast-Asian pepper diversity.

### Genomic Scans Reveal Selection for Culinary and Aesthetic Traits.

The *Capsicum* domestication syndrome includes the transition of fruit position from deciduous erect to nondeciduous pendant (28), changing in the degree of the pungency of fruits, an increased fruit weight and shape variation, and shortening transition to flowering. As observed within genomic diversity data, we found a phenotype-based germplasm stratification according to the origin regions (Fig. 2). These findings highlighted a set of traits that drove the spread and differentiation in pepper. To date, the cultivated types contain both small pungent peppers used as spice or condiments as well as large-fruited and sweet types which are the most economically important worldwide (6, 29, 30). Therefore, the differentiation of gene pools is linked to several factors including cultures, consumer preferences, and country economies. This explains how additional centers of diversification occurred within the major producer countries (e.g., North America and Asia) where intensification of breeding programs allowed the release of new cultivars.

Genome-wide association studies (GWAS) detected several notable marker–trait associations for key traits under selection, several of which correspond to known quantitative trait loci (QTLs) and fall near genes with documented functions (Fig. 4 and *SI Appendix*, Table S7). Fruit pungency was primarily associated with a peak on the pepper chromosome P2 spanning 142 to 150 megabase pairs (Mbp) (Fig. 4*A*), falling 2.5 Mbp upstream from the acyltransferase *AT3* gene, previously implicated in capsaicin biosynthesis (31). Among closest candidates, a *PFP-BETA* encodes for a pyrophosphate phosphotransferase involved in the first step of glycolysis that produces pyruvate, which (via a four-step pathway) contributes to the synthesis of valine (32), a vital precursor of capsaicin—a possible mechanism for future investigation. A local depression in Tajima’s D occurs in association with this distal P2 region (Fig. 4*B*), a sign of possible purifying selection in favor of a selected number of pungency alleles, in accordance with regional culinary desires.

Pedicel and fruit position, which are highly correlated and responsible for berry position from fruit set to ripeness, (*SI Appendix*, Fig. S13), were found strongly associated with markers on P12 in the vicinity of *Protein EARLY FLOWERING 4* (*ELF4*)-like (33), whose *Arabidopsis thaliana* ortholog plays a role in circadian regulation, which in turn controls the flower position through day–night cycles. Moreover, for pedicel position, an additional associated peak was identified within the gene *FAB1B*—which can affect pollen traits—and on P2, close to the floral promoter *CONSTANS*, which has a broad range of documented roles (34), including in day length perception and calyx morphology. Neither of them presents an obvious mechanistic interpretation at this stage.

In addition, we found candidates for traits not yet fully explored in pepper. The number of pedicels per axil (Fig. 4*C*), a trait that might affect yield potential via an increase in fruit number, was most strongly associated with a GWAS peak on P6 in a region likely under selective pressure. The two nearest annotated genes, a hypothetical but unclassified protein and a SRSF protein kinase, do not lend themselves to speculation on the mechanism. However, the strength of the association coupled with the agricultural implications of the trait should make this observation a prime candidate for further research by breeders and geneticists. Finally, strong associations were found for flower annular constrictions on P1, although none appears to be linked with known functional genes, and no indication of selective sweep or balancing selection is evidenced on this chromosome (Fig. 4*D*).

GWAS highlights how a large factor in pepper’s initial appeal was certainly its pungency, suggesting putative regions for selection in correspondence of some primary traits that has driven the selection. By confirming established associations, we provide proof of the reliability of genebank records for discovering candidate genes. This suggests a key position of genebanks as a central repository of germplasm and related big data (genomics and phenomics) toward future gene discovery and crop improvement.

## Conclusion

Our research represents a case study in the exploitation and in-depth analysis of genetic data from genebank collections to yield information on expansion routes of the most economically important pepper species (*C. annuum*). Historians, archaeologists, and ethnobotanists have discussed evidence of many migration pathways including 1) longitudinal migration through the Americas, 2) three out-of-America pathways (to Eurasia directly via Spanish/Portuguese trade routes, into Eurasia via Africa and the Indian Ocean via Portuguese and Arabic trade routes, and directly to South/Southeast Asia) through pre- or post-Columbian trans-Pacific trade routes, 3) overland migration throughout Eurasia, and 4) transatlantic slave trade connecting America, Europe and Africa. Our investigation using the ReMIXTURE method shows that the primary similarities between peppers collected in these regions accord with all these pathways, except for the direct Asia–America link, a hypothesis on which the analyses are somewhat equivocal. Mesoamerica, East/South/Southeast Asia, and Africa are all notable for large proportions of region-unique peppers indicating their status of diversification center. The broad overlapping clines that are formed by each region’s peppers suggests that crossing between locally favored varieties and new acquisitions have frequently occurred. As our GWAS and selection scan results confirm, a large factor in pepper’s initial appeal was certainly its pungency, especially in nontropical Europe where hot spices were rare and imported black pepper (*Piper nigrum*) could fetch demanding prices. This study shows how genomics applied to a horticultural crop can offer opportunities for PGR management and provide valuable insight on the conserved germplasm resources. In fact, genetic data stored in genebanks confirm that pepper, thanks to its flexible features (easily preserved and transportable in dried form, needed in moderate quantity to enrich dishes, easy to produce, and wide scale) has been spread along with the very earliest intercontinental traders, being among the very earliest examples of a globally traded, mass-market, consumer-discretionary good.

## Materials and Methods

### Germplasm Collection.

The germplasm studied here was comprised of a total of 10,038 pepper accessions. Based on genebank passport information, these accessions originated from 130 countries across five continents and represented 9,689 accessions from the domesticated species, 83 from seven wild species, and 266 (2.5%) had an undetermined species status. Details of species, genebanks, and passport information provided by provider curators are reported in *SI Appendix*, Table S1 and Dataset S1.

### DNA Isolation and Library Construction.

Genomic DNA was extracted from 100 mg fresh leaf tissue collected from individual plants for each accession. DNA extraction was performed either with the DNeasy Plant Mini Kit (QIAGEN) or the Sbeadex maxi plant kit (LGC Genomics). DNA quantity and quality parameters were assessed using both spectrometry (ND-1000; NanoDrop, ThermoScientific) and fluorometry (Qubit 2.0 Fluorometer, Invitrogen) methods. Samples with 260/280 and 230/260 ratios ranging between 1.8 to 2.2 and 1.8 to 2.0, respectively, and with a less than twofold deviation between fluorimetric and spectrophotometric readings were subjected to genotyping-by-sequencing. As controls, the reference *C. annuum* cv. CM334 (originating from a unique seed batch provided by INRAE) was included in each library preparation (total: 224 controls). For complexity reduction, a two-enzyme protocol using *Pst*I (CTGCAG) and *Msp*I (CCGG) was used (35). DNA sequencing library preparation was performed essentially as described by Wendler et al. (36). Size selection of 250- to 600-base pairs (bp) (insert size 130- to 480-bp) fragments was done using a Blue Pippin (Sage Science). Library pools were quantified using an Agilent Tape Station and qPCR. In typical experiments, 188 individually barcoded were multiplexed and sequenced using an Illumina HiSeq2500 platform generating 1 × 107-bp single-end reads version 3 chemistry (Illumina).

### Read Alignment and Variant Calling.

Quality of sequencing reads was filtered using FastQC (37). The low-quality bases and adapter sequences were removed using cutadapt (38). The 10,280 million trimmed reads generated from sequencing of 10,262 samples (germplasm collection plus CM334 control accessions) (*SI Appendix*, Table S8) were then aligned to reference genome sequence *C. annuum* CM334 version 1.6 available at http://peppergenome.snu.ac.kr (39) using BWA-MEM version 0.7 (40) and converted to binary alignment map format using SAMtools (41). The alignments from each sample were sorted and indexed using NovoSort and used for variant calling using SAMtools/BCFtools version 1.9 (42). The pipeline was run with minimum quality cutoff (q) of 20 and keeping other parameters to default. Reads showing a (Q) ≥ 30 were mapped against reference genome (*SI Appendix*, Table S8). A 527,474 biallelic SNPs matrix was obtained with minimum QUAL ≥ 40; minimum read depth for homozygous call ≥ 2; minimum read depth for heterozygous calls ≥ 4. For downstream analysis, SNP sites were retained with minor allele count (mac ≥ 50), heterozygosity level (<5%), and missing data (20%). Imputation of missing genotype calls in the SNP matrix was done using the FILLIN algorithm implemented in TASSEL 5 (43). Functional annotation of the identified variants associated genes was performed using SnpEff (version 3.1) (http://snpeff.sourceforge.net/). The commands were run in parallel to reduce computational time using GNU parallel (44).

### Identification of Sample Duplication.

In order to estimate the degree of duplications, allele matching was calculated as provided by an absolute percent IBS coefficient between all individuals. IBS was calculated using the snpgdsIBSNum function of SNPRelate (45). The IBS threshold value (0.0001) was imputed based on the IBS values of the CM334 controls used in the analysis. Hierarchical cluster analysis was performed as an additional means of visualizing relatedness of accessions based on the pairwise comparison. A stringent z-threshold of 90% was used to define clusters of accessions.

### Genomic Diversity and Phylogeny.

The analysis of genomic diversity and genetic relationships were inferred using several approaches. To gain a purely descriptive illustration of the genetic diversity in the sample and its relationship to agronomic traits, we performed PCA with SNPrelate. To evaluate the degree of genetic isolation among pepper species, we estimated Weir and Cockeram’s weighted *Fst* for all pairwise combinations of species using VCFtools version 0.1.17 (46). To investigate possible signatures of selective sweeps, we estimated Tajima’s D, which detects local discrepancies between nucleotide and haplotype diversity, suggestive of departures from mutation–fixation equilibrium under a neutral drift model, using VCFtools version 0.1.17 in nonoverlapping 1-Mb windows.

We tested two model-based approaches (ADMIXTURE and phylogenetic tree inference) in an attempt to assess the degree to which models that imply some form of genetic structure were suitable to the data. We ran ADMIXTURE version 1.23 (47) with the following parameters: number of subpopulations (K) ranging from K = 1 to 15, 10-fold cross-validation (CV) with five iterations, 1,000 bootstrap replicates to estimate parameter SEs, and 500 random seed for reproducibility. CV scores were used to determine a somewhat suitable value for K. Individuals were tentatively assigned to one of the K populations if/when its membership coefficient in that group was ≥0.50. We then generated a dendrogrammatic representation of the population’s structure in a maximum likelihood (ML) framework, using IQ-TREE (48). Branch supports were obtained with the ultrafast bootstrap (49). Comparison between ML trees was assessed using the Robinson–Foulds distance calculated with ETE 3 (50).

In order to present the data in a way amenable to elucidating the exchange and sharing of pepper varieties among cultures, we developed the ReMIXTURE (“Regional Mixture”) method, a purely descriptive visualization method that asks, intuitively, for a series of nominated regions r=1..R in turn, “how much overlap do the taxa from this region tend to have with those grown in each other region?” We aimed to produce a measure of overlap that is intuitive to interpret and which meets the criteria 1) that any preferential sharing of accessions between regions in either direction should reasonably be expected to increase the overlap between them, 2) that the measure should also reflect the degree to which a region’s accessions are not similar to accessions in other regions (i.e., that some kind of self-overlap is measurable and makes sense), 3) that the measures are specific to a focal region and need not be symmetrical between regions, and 4) that the measure should be resistant to biases caused by different sample sizes across regions. We therefore calculate overlap between regions as follows. The set of samples from a region are denoted $s_{r\in R}$. The set of all samples across all regions is denoted S. A matrix of distance measures $D_{s\_1,s\_2}$ is defined, containing the IBS distances between individual s_1 and s_2, for all pairwise combinations of individuals s_1, s_2 ∈S. We iterate over i=1..I. At each iteration:

1) A matrix of counts $C_{r_{1}=1..R,r_{2}=1..R}^{i}$ is defined with all entries initialized to zero.
2) A subset of S, $S^{i,N}$is defined, which contains *N* random individuals from each region in R. *N* is a constant, chosen to be significantly smaller than the number of samples in the most sample-poor region.
3) For each sample $s_{r}^{1..N}\in S^{i,N}$, the closest nonself neighbor $s{'}_{r'}^{1..N}\in S^{i,N}$ is identified (in each case, this can be found as the individual corresponding to the lowest nondiagonal entry in the row$D_{s_{r},.}$). $C_{r,r'}^{i}$ is incremented by 1.

The global relative overlap matrix O is obtained by elementwise summing $C^{i}$ over all i, then normalizing each row to give proportions. The entries now meet criteria 1 to 3 above, with the magnitude of the diagonal entries giving an indication of self-overlap, fitting criterion 2.

A bootstrapping procedure can be used to estimate CIs for the entries of O, by sampling m random iterations from $C^{i}$, P times, calculating an overlap matrix $O^{p=1..P}$ for each resampling, and finally calculating the average squared deviation of each entry in the Op's from the corresponding entry in the global relative overlap matrix O.

The ReMIXTURE approach was run for all *C. annuum* accessions, excluding those from Oceania which had very low sample numbers, using the IBS matrix calculated as described for the analysis of duplicated samples, with the following parameters: *I* = 2,000, *N* = 54 (set to one-half the number of samples in the most sample-poor region), *m* = 1,500, and *P* = 1,000. Details of the R (51) implementation of ReMIXTURE used in this publication (including visualization scripts) are in *Data Availability*. The implementation relies significantly upon the R packages data.table (52), ggplot2 (53), ggspatial (54), rnaturalearth (55), and pheatmap (56).

### GWA Analysis.

Phenotypic data for 21 qualitative/pseudoqualitative descriptors for plant, flower, and fruit trait categories recorded by each genebank during multiplication cycles following the International Plant Genetic Resources Institute (IPGRI)/Bioversity (57) protocol for the genus, were analyzed, including three morphological plant traits, six flower, and 12 fruit traits (*SI Appendix*, Table S9). Before proceeding with the analysis, all data have been reviewed removing any inconsistencies (e.g., traits not registered equally) and outliers. This led to the establishment of a core set of 2,059 *C. annuum* accessions representing the variation of the whole species gene pool (*SI Appendix*, Fig. S14) and without any missing data for 21 phenotypic observations (Dataset S4).

In order to widen the genetic variants detected in GWAS such as major rearrangements, insertions, and deletions, reference-free GWA analysis was implemented using a *k*-mer GWAS pipeline (58). This pipeline uses k-mers, as markers for GWA analysis. Firstly, the 31-bp *k*-mer sequences were identified from the quality trimmed reads from each sample using KMC (version 3.0). The software was run with minimum read count threshold of 2 and keeping other parameters to default. The *k*-mers from all the samples were then compared to generate a k-mer presence/absence matrix which was further filtered with minor allele frequency (MAF) ≥ 0.02 and minor allele content (MAC) ≥40. The kinship matrices of relatedness between the samples were calculated based on the filtered k-mer matrix and using EMMA package (59) with default parameters. Finally, GWAS was carried out using mixed linear model (MLM) from package GEMMA (version 0.96) (60) for each trait separately. The *P* value threshold was determined based on permutations of phenotype. For visualization of the results, the top 1 million k-mers for each trait were extracted from GWA study and mapped against the reference genome sequence *C. annuum* CM334 version 1.6, and positions of uniquely mapped *k*-mers were retrieved. The Manhattan plots for each trait were generated using qqman R package (61).

## Supplementary Material

Supplementary File

Supplementary File

Supplementary File

Supplementary File

Supplementary File

## Data Availability

All study data are included in the article and/or supporting information. The raw data sequences (FASTQ files) are available on the European Nucleotide Archive (ENA) under the accession number BioProject PRJEB45375. The source codes used to perform ReMIXTURE analysis (including visualization scripts) are available on GitHub (https://github.com/mtrw/ReMIXTURE).
