# Supplementary material for: Global range expansion history of pepper (Capsicum spp.) revealed by over 10,000 genebank accessions
Source: Proc Natl Acad Sci U S A. 2021 Aug 16;118(34):e2104315118. doi: 10.1073/pnas.2104315118 (PMC8403938; doi:10.1073/pnas.2104315118)
Supplement: Supplementary File [file pnas.2104315118.sapp.pdf]

PNAS
www.pnas.org

Supplementary Information for

Global range expansion history of pepper (*Capsicum* spp.) revealed by over 10,000 genebank accessions

Pasquale Tripodi, Mark Timothy Rabanus-Wallace, Lorenzo Barchi, Sandip Kale, Salvatore Esposito, Alberto Acquadro, Roland Schafleitner, Maarten van Zonneveld, Jaime Prohens, Maria José Diez, Andreas Börner, Jérémy Salinier, Bernard Caromel, Arnaud Bovy, Filiz Boyaci, Gancho Pasev, Ronny Brandt, Axel Himmelbach, Ezio Portis, Richard Finkers, Sergio Lanteri, Ilan Paran, Véronique Lefebvre, Giovanni Giuliano, Nils Stein

Pasquale Tripodi, Nils Stein
Email: pasquale.tripodi@crea.gov.it, stein@ipk-gatersleben.de

This PDF file includes:

Figures S1 to S14
Tables S1 to S9
Legends for Datasets S1 to S4

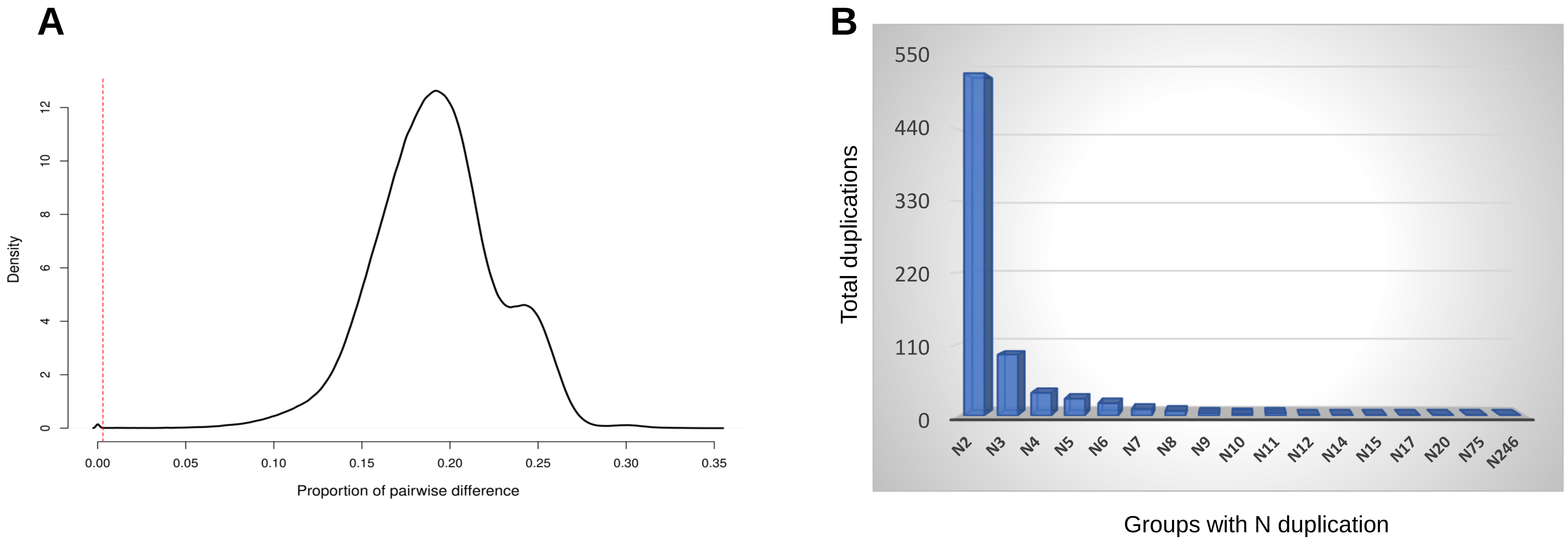

Fig. S1: Identification of genebanks potential duplicates**. A)** Distribution of pairwise homozygous difference in identity by state values between pairs of samples. The red line (0.0001%) marks the threshold for calling sample pairs potential duplicates. **B**) IBS groups carrying duplicated accessions across genebanks; on the X-axis are reported the number of duplications for each group (e.g. 2, 3 etc.), on the Y-axis the total number of groups identified containing two or more duplications.

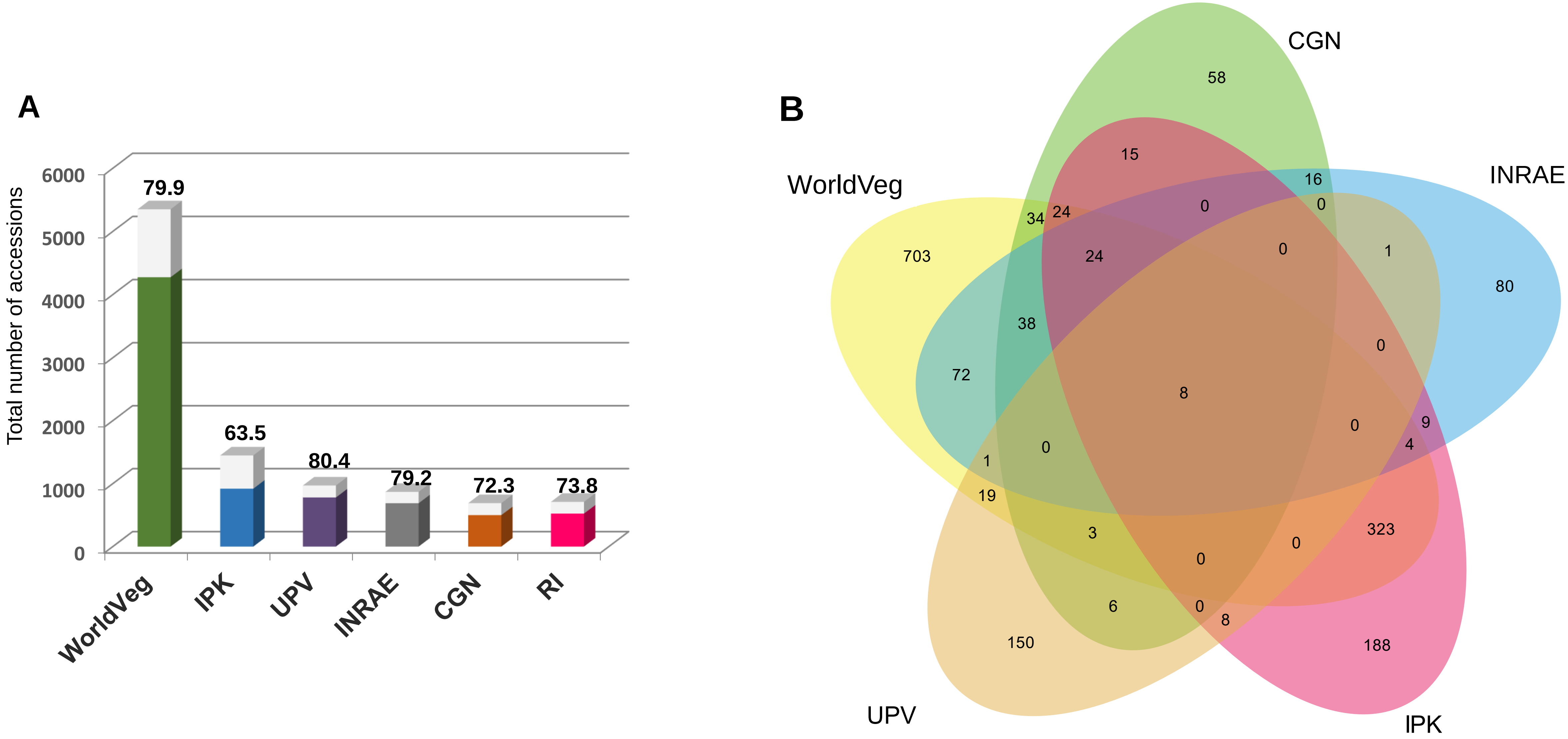

Fig. S2: Germplasm shared among genebanks**. A)** Bar plot showing the ratio of unique/duplicated accessions in the whole collection for germplasm providers. Colored and white bars indicate the proportion of unique and duplicated accessions for each genebank, respectively. The percentage of unique accessions is reported on the top of each bar. On the Y-axis is reported the total number of accessions provided. **B**) Venn diagram representing duplicated accessions within and among the five main international genebanks holding *Capsicum* germplasm. WorldVeg = World Vegetable Center; IPK = Leibniz-Institut für Pflanzengenetik und Kulturpflanzenforschung; UPV = Universitat Politècnica de València Germplasm Bank; INRAE= Centre de Ressources Biologiques Légumes de l'Unité de Génétique et Amélioration des Fruits et Légumes; CGN = Centre for Genetic Resources; RI = Research Institutes (ARO, CREA, UNITO, BATEM and MVCRI; for details see Table S1).

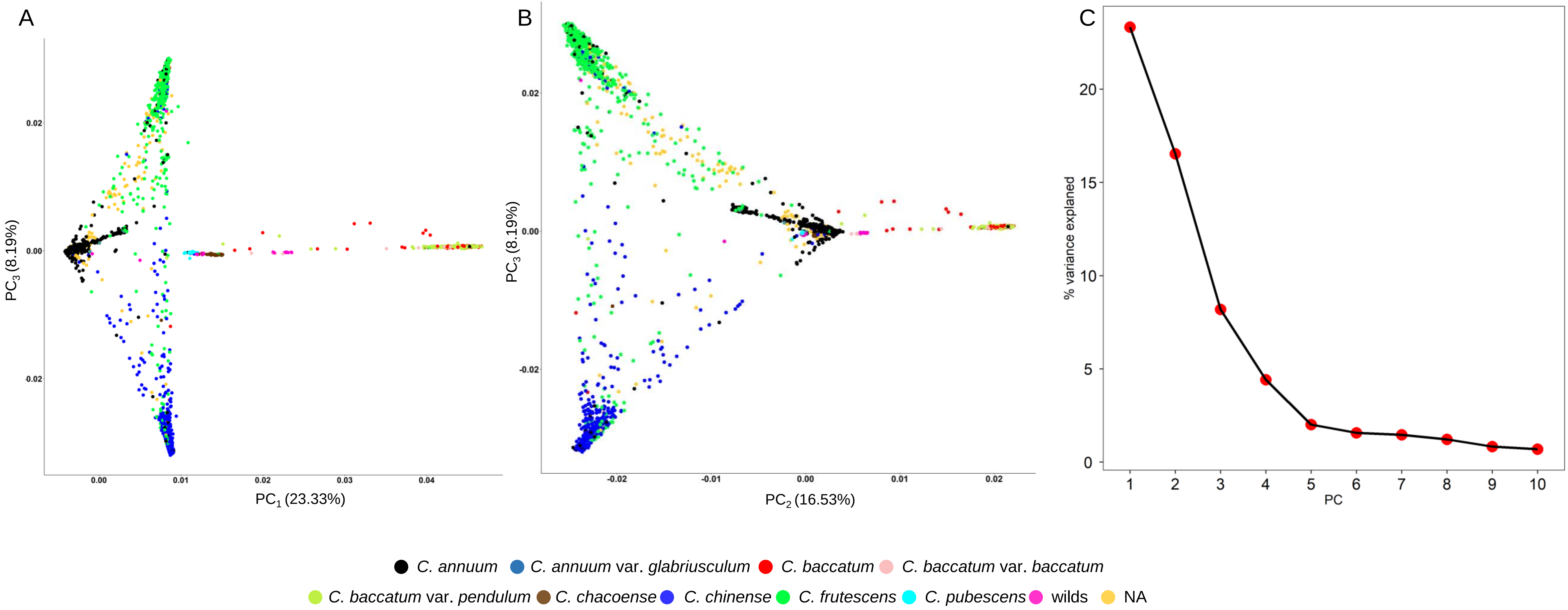

Fig. S3: PCA plots considering showing genetic diversity among the full (undeduplicated) data set of 10,262 *Capsicum* accessions (10,038 samples and 234 control accessions). A) 1st and 3rd components; B) 2nd and 3rd components; C) plot showing variance captured in the first 10 PCs. Samples are colored according to species (palette on the bottom)

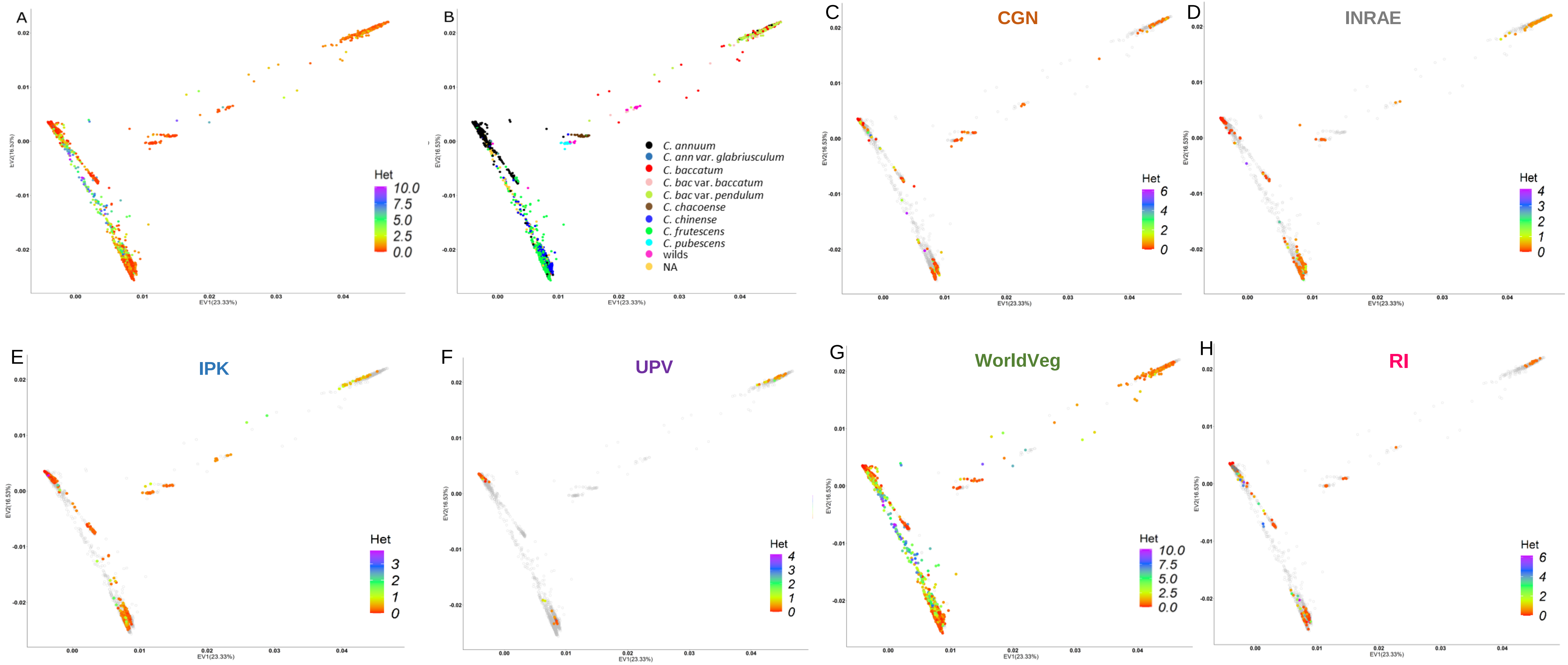

Fig. S4: Heterozygosity of the collection studied. Levels of heterozygosity on the first two components (A) according to *Capsicum* species distribution (B). Details for main genebank (C-G), and main research institutes (RI; see Fig S2) holding *Capsicum* germplasm (H). Average, minimum and maximum heterozygosity values are reported in Table S6

Fig. S5: Genetic structure of *C. annuum* collection**. A)** Model-based admixture analysis of 7.848 *C. annuum* genotypes with 26,566 SNP markers in the case of nine clusters (K). The colored blocks correspond to the different cluster. **B**) Cross validation error (CV) for ADMIXTURE analysis in cultivated pepper (*C. annuum*). Runs of K ranging from 1 to 15; the most likely number of subcluster were at K = 9; **C**) Map showing the geographical distribution of accessions according to K groups. The color for each country indicates the group (K) of belonging. Color dots indicate the presence of accessions in different groups. The two most represented K are reported, as an example, for USA, accessions cluster mainly in K = 3 (most represented cluster) and K =4. The main cluster (K=1) comprises Eastern European and Middle Eastern accessions; at K = 2 are grouped accessions retrieved from South and Southeast area(mostly India, Indonesia, Malaysia and Thailand); K=3 comprises accessions mostly collected in Italy, Spain, France, and the Netherlands, as well as those from China and North America. This cluster includes mostly the commercial sweet pepper types with blocky, bell, and rectangular shape; K=4 includes accessions from the East regions principally Korea, Japan, and China; K=8 includes accessions retrieved from the Mediterranean basin and mostly collected in Italy and Spain, as well as several accessions from Africa and South America; K = 5 and K = 9 mostly include Mesoamerican accessions, whereas K = 6 and K = 7 are the smallest clusters including miscellaneous accessions of different origin**. D**) Pie charts indicating the proportion assignment to each genetic subgroup (K) for each region, in brackets are reported the number of accessions for the considered regions. Accessions from Oceania (13) are admixed and the remaining 465 accessions have incomplete passport data.

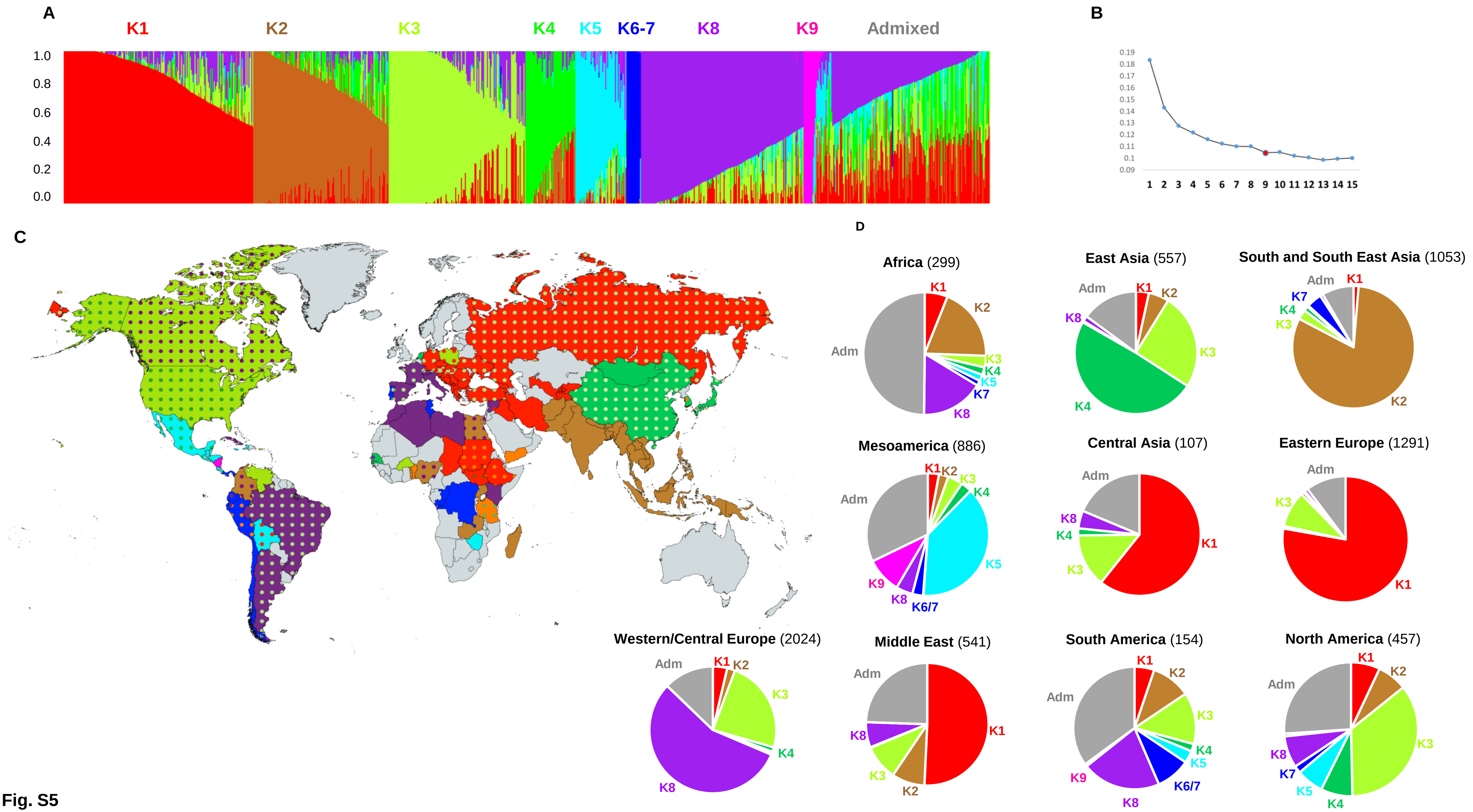
A
K1
K2
K3
K4
K5
K6-7
K8
K9
Admixed
1.0
0.8
0.6
0.4
0.2
0.0
B
0.19
0.18
0.17
0.16
0.15
0.14
0.13
0.12
0.11
0.1
0.09
1 2 3 4 5 6 7 8 9 10 11 12 13 14 15
C
D
Africa (299)
East Asia (557)
South and South East Asia (1053)
Mesoamerica (886)
Central Asia (107)
Eastern Europe (1291)
Western/Central Europe (2024)
Middle East (541)
South America (154)
North America (457)
Adm
K6/7
K7

Fig. S5

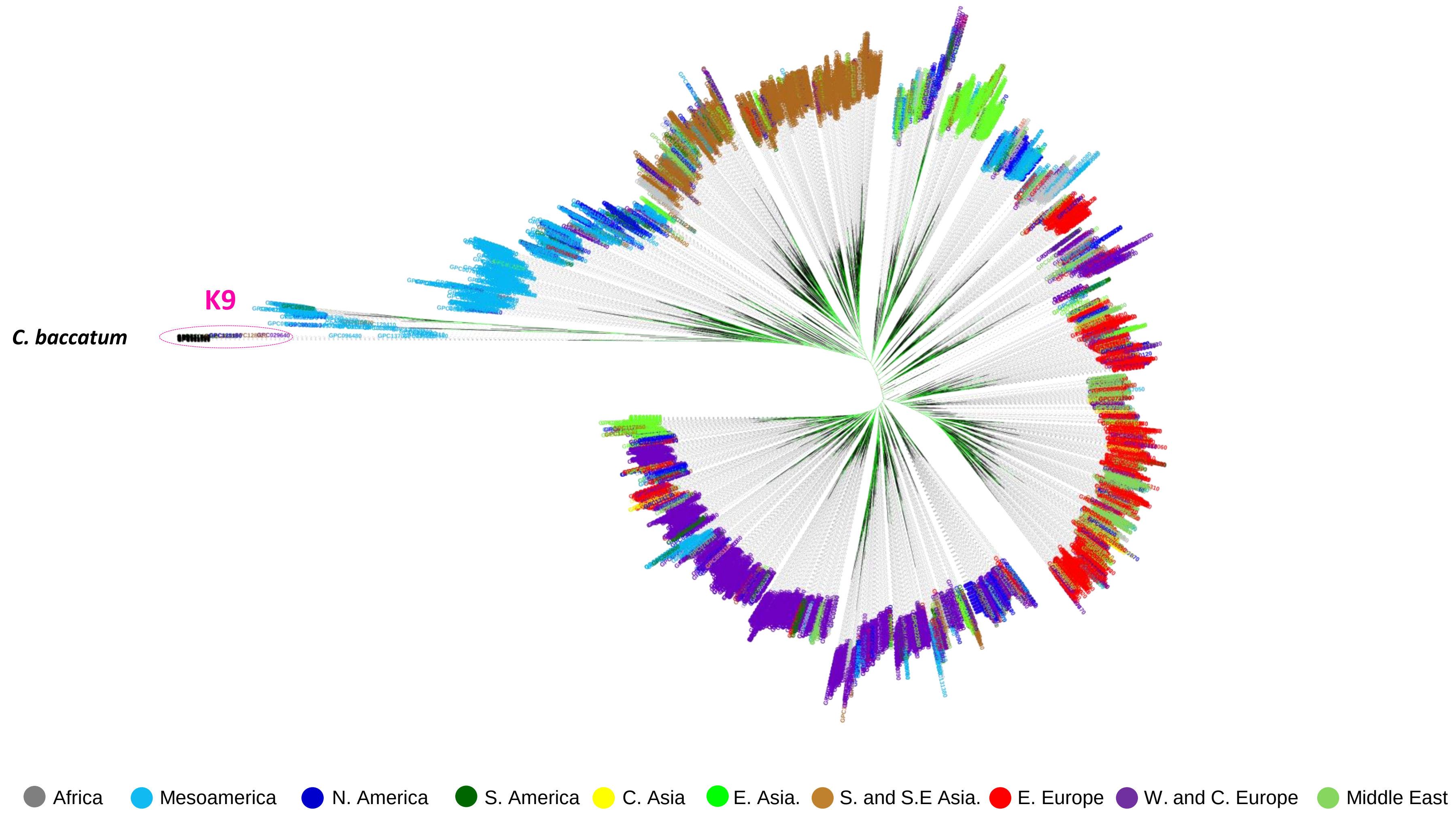

Fig. S6: Maximum likelihood phylogenetic tree (unrooted) based on 26,566 markers for 7382 *C. annuum* genotypes and 10 *C. baccatum* accessions as outgroups. Entries are coloured according to geographical provenance indicated by colour chart in the legend. The tree shows the European genepool clearly distinct from the Asian and Mesoamerican ones with only a few exceptions. Eastern European accessions are intermingled with the germplasm from the Middle East. A main branch includes the Asian, African, and Mesoamerican germplasm, although the accessions within were grouped in single clusters representative of the respective areas. The northern American accessions are interspersed within the Central American and European groups. (C. = Central; E. = Eastern; N. = North; S. = South; W. = Western).

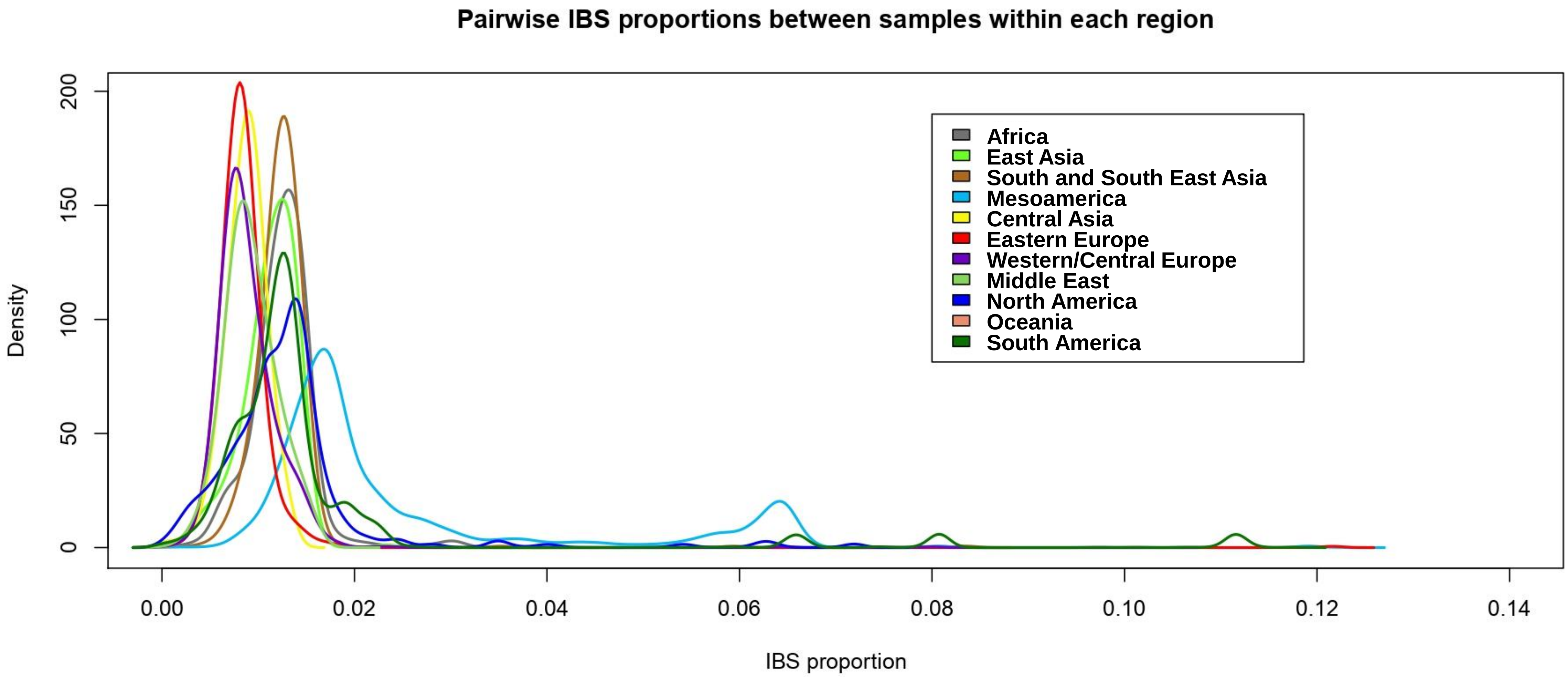

Fig. S7: Distribution of pairwise identity-by-state proportions between *Capsicum annuum* samples from each global region (Gaussian kernel density, bandwidth=0.001), showing larger founding diversity among the Mesoamerican samples, a lower degree in North and South America, moderate diversity in East/South-Southeast Asian samples, and lower diversity in the Middle East, Central Asia, and Eastern/Western-Central Europe, a probable effect of genetic bottlenecking resulting from local cultural and culinary preferences.

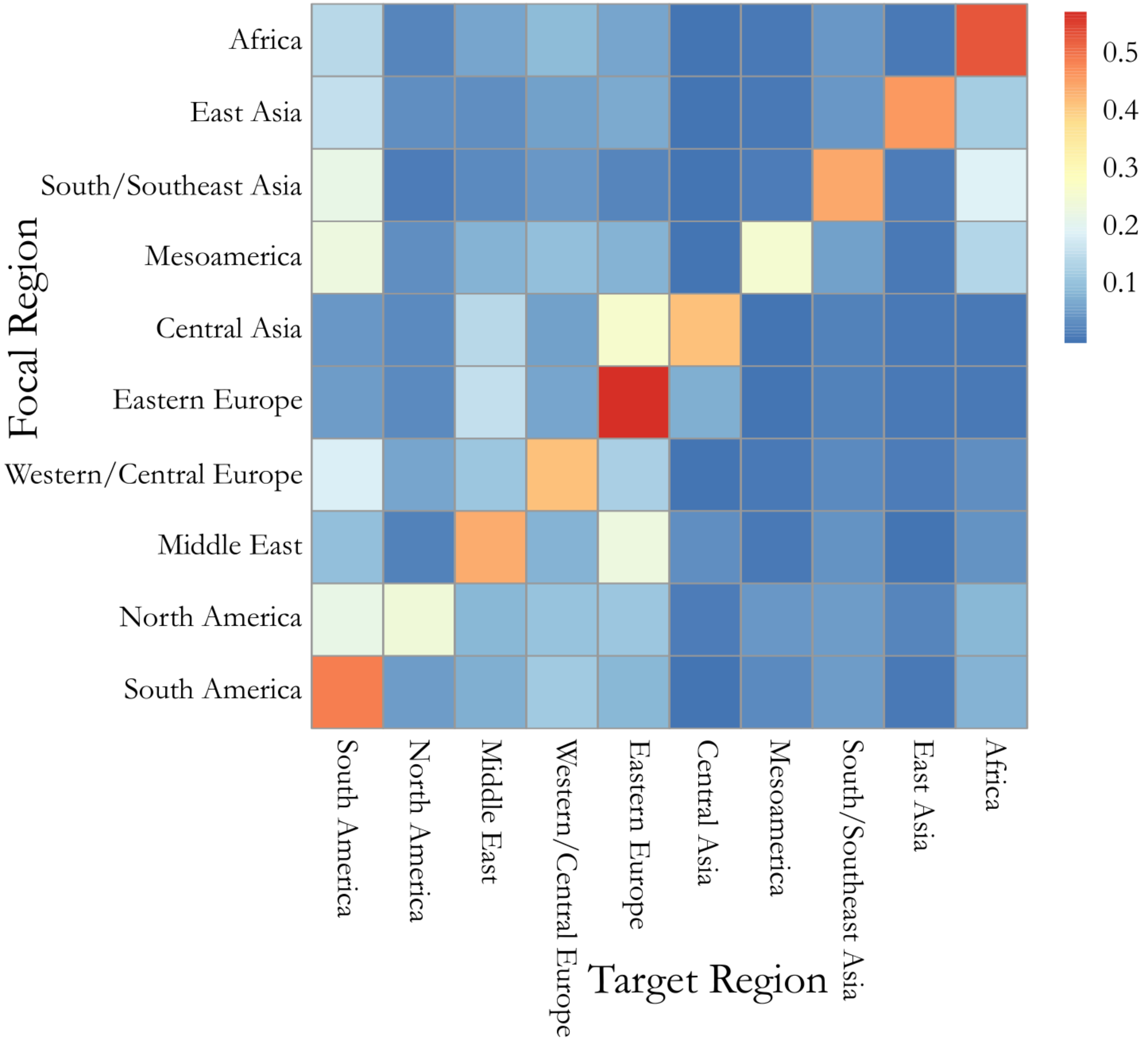

Figure S8. RGO values for 10 global regions, produced using the ReMIXTURE procedure (see text; Methods). Intuitively, RGO indicates the degree to which pepper diversity in the target region "overlaps" the diversity of the focal region. Oceania is not included due the low number of accessions.

A B

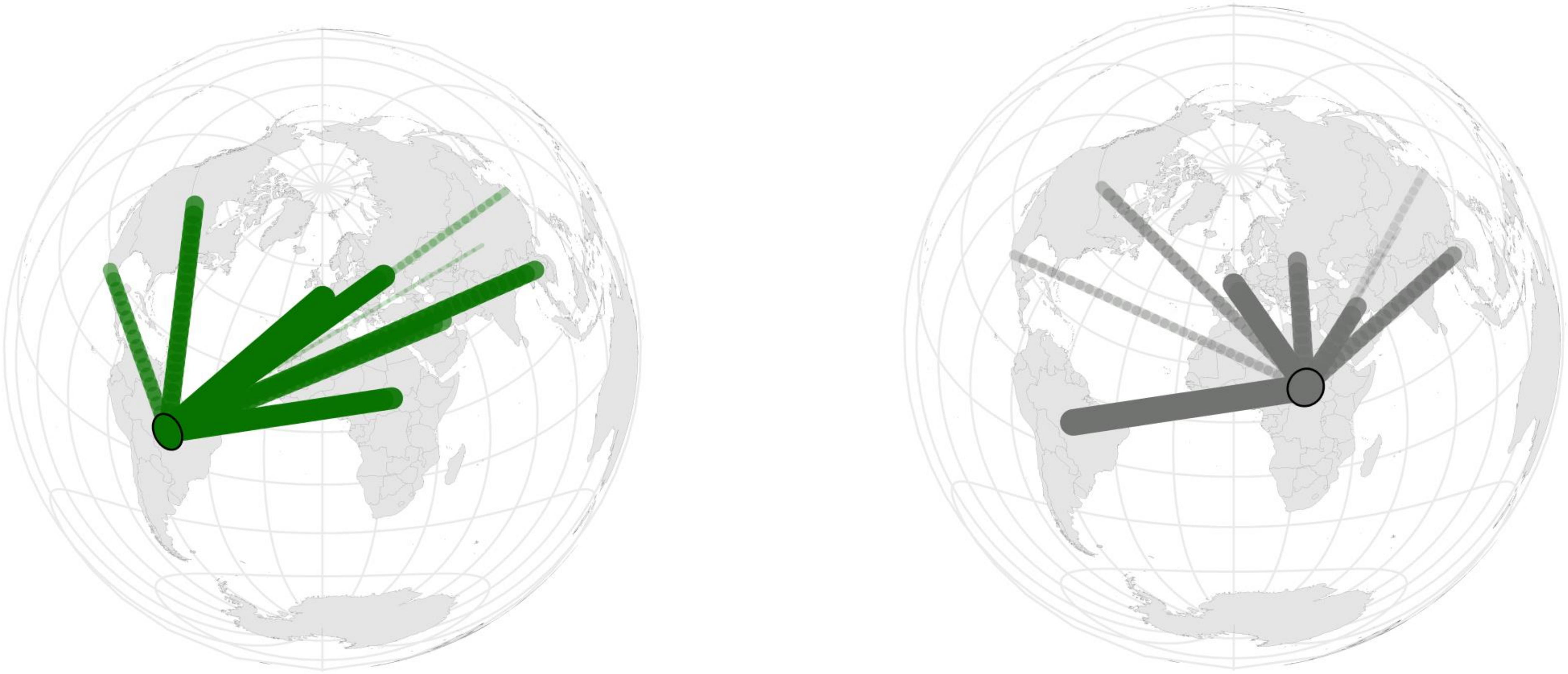

Fig. S9: Details of RGO’s among regions: A) South America, B) Africa

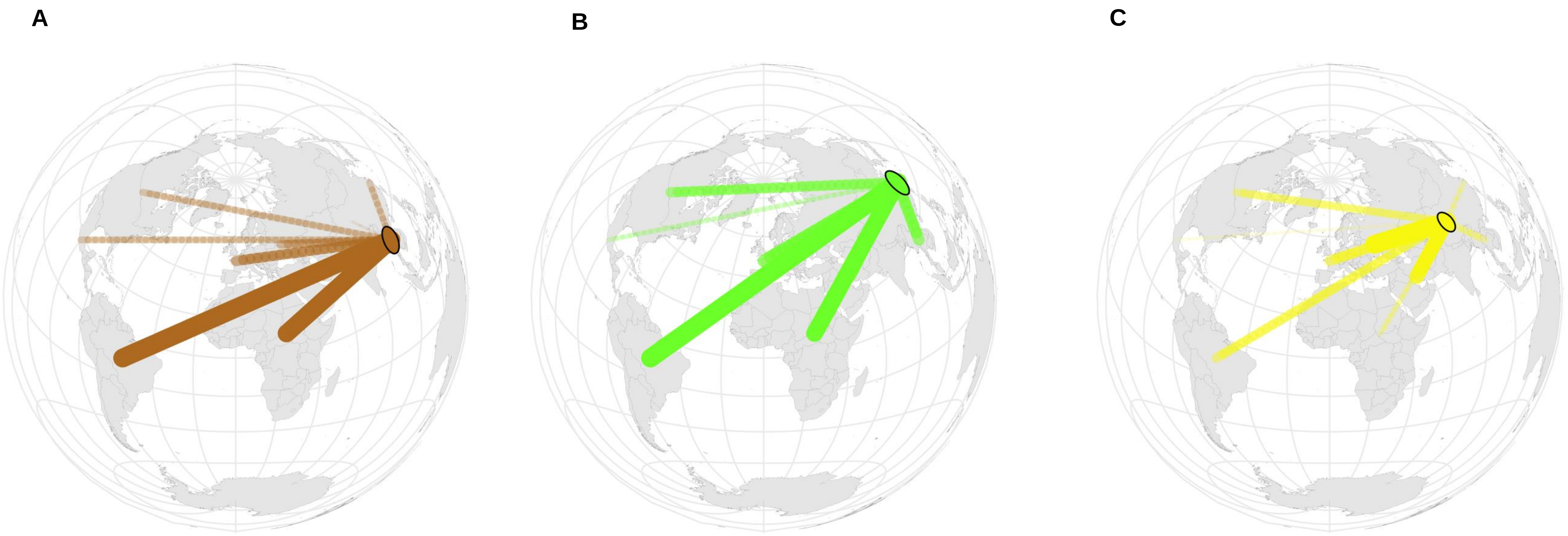

Fig. S10: Details of RGO's among regions: A) South and South East Asia; B) East Asia: C) Central Asia

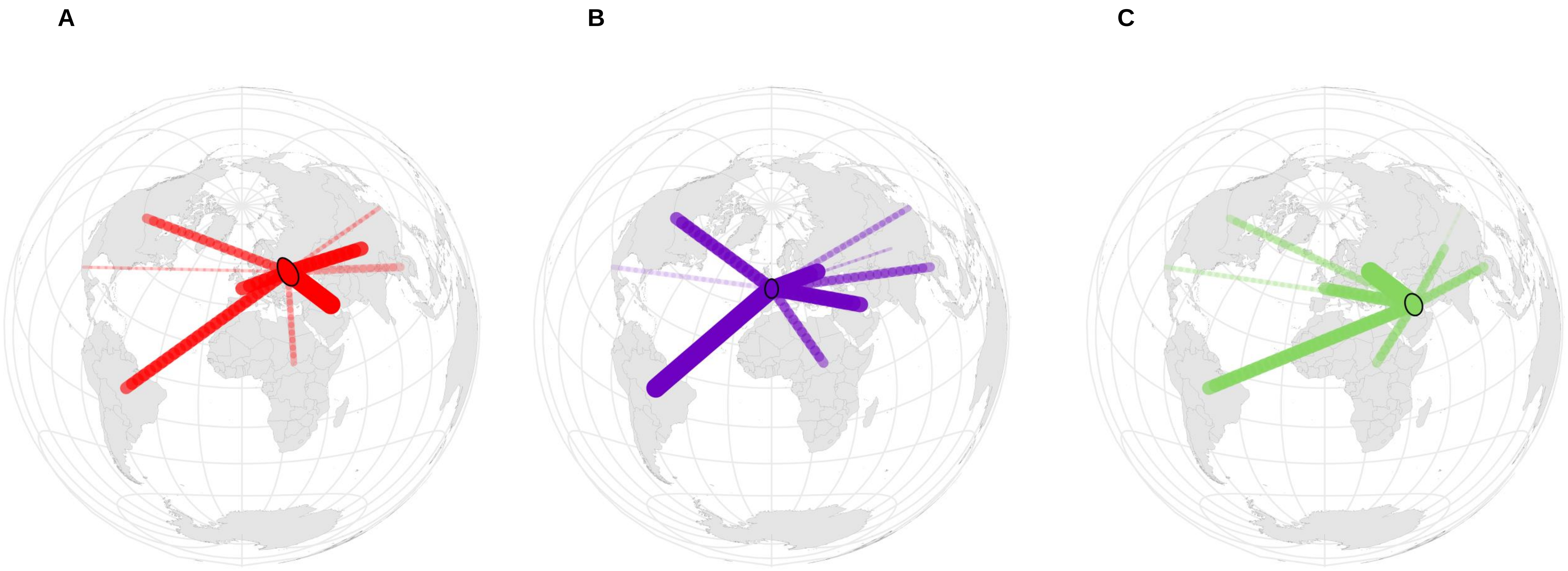

Fig. S11: Details of RGO’s among regions: A) Eastern Europe, B) Western and Central Europe, C) Middle East

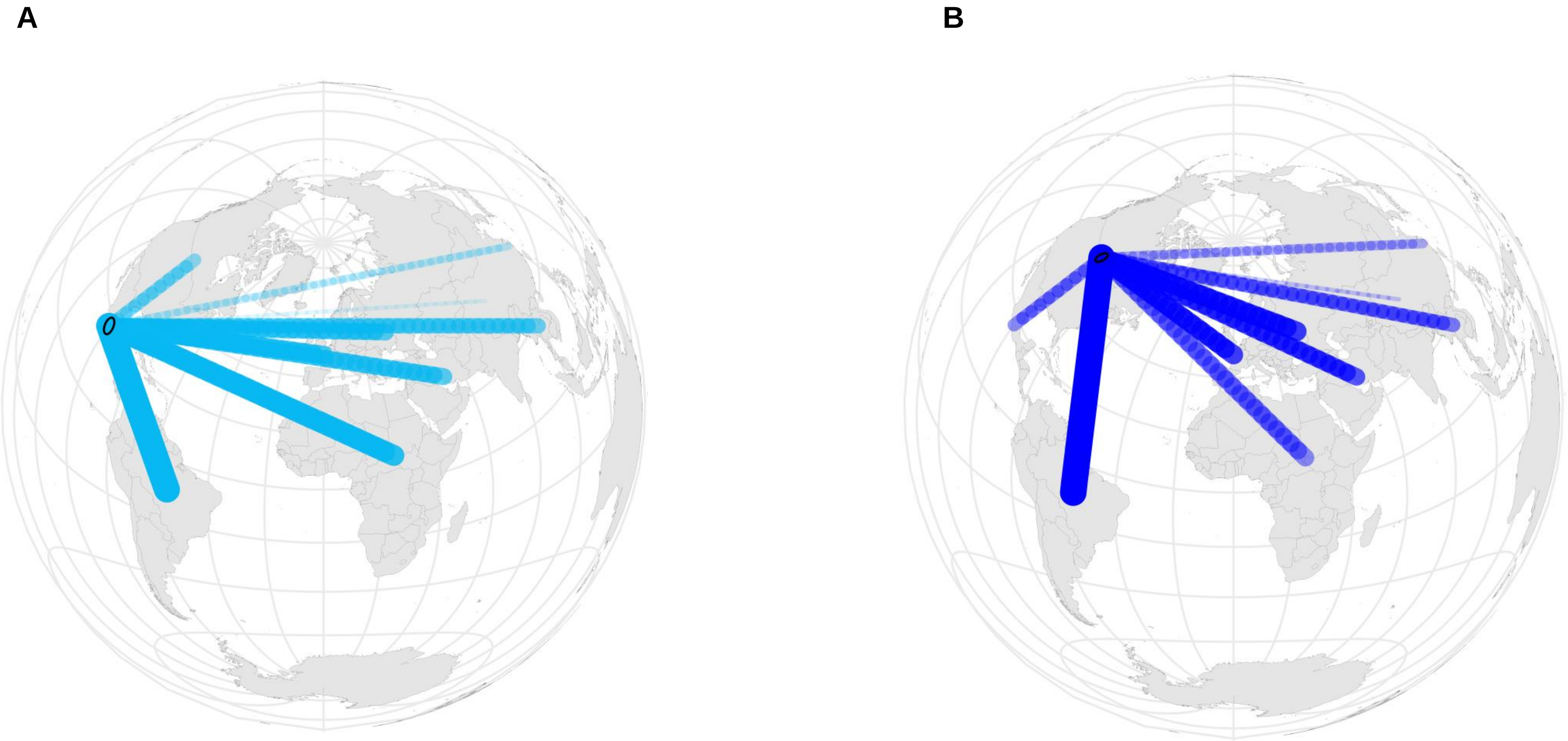

Fig. S12: Details of RGO's among regions: A) Mesoamerica, B) North America

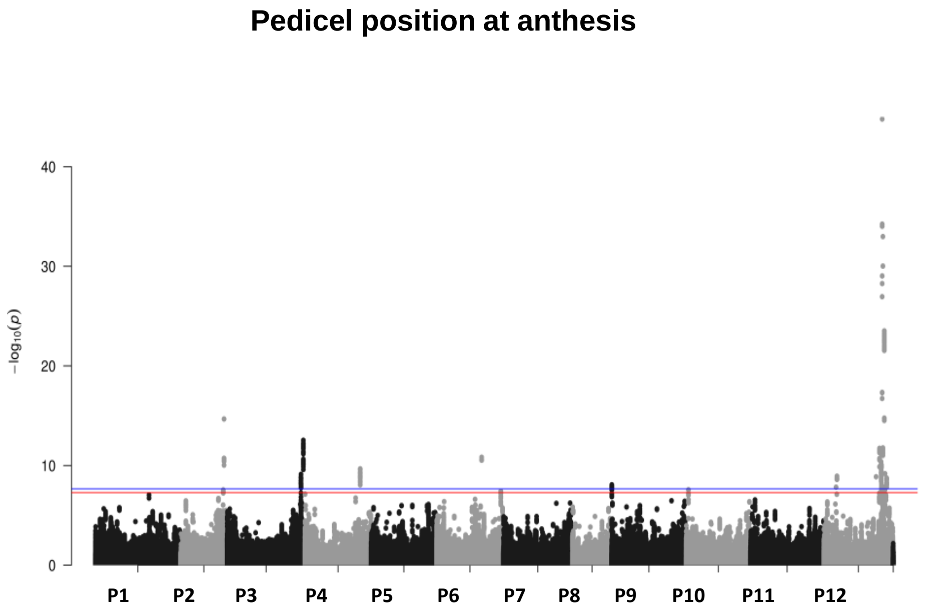

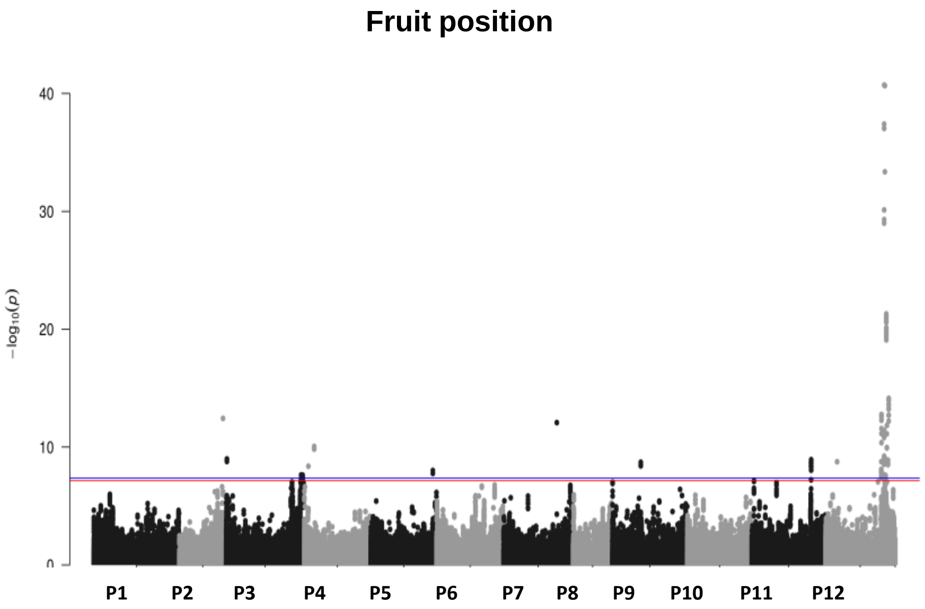

Fig. S13: Manhattan plots showing marker-trait associations for pedicel and fruit position in *C. annuum*. The blue line represents the genome-wide significance threshold (1.60E-08) and the red line represents the suggestive association threshold (4.00E-08).

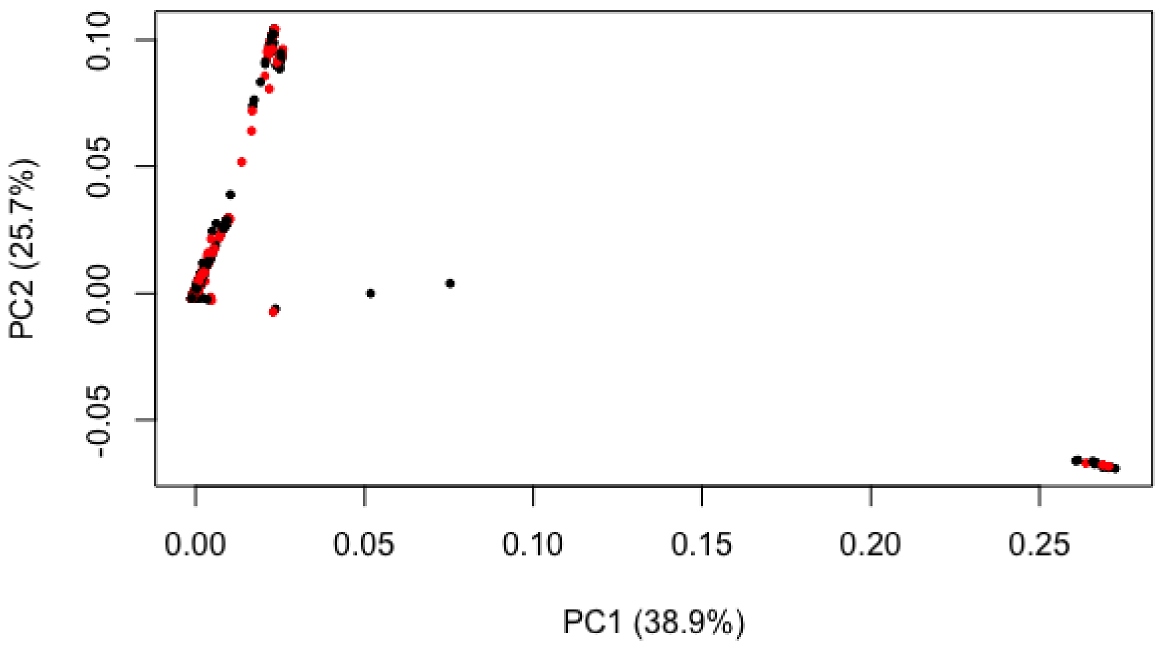

Fig. S14. MDS plot showing the distribution of 2059 *C. annuum* individuals composing the core collection for GWAS (red dots) on a total of 7848 accessions.

Table S1. Summary of plant material provided by genebanks within each *Capsicum* species in the present study.

Species	N°	WorldVeg	IPK	UPV - COMAV	INRAE	CGN	ARO	CREA	UNITO	BATEM	MVCRI
**Domesticated**											
C. annuum var. *annuum*	7848	4084	1138	928	637	470	215	199	92	48	37
C. frutescens	745	459	170	8	38	41	21	8			
C. chinense	646	310	51	9	98	115	31	30	2		
C. baccatum (var. unknown)	163	85	2	18	46		8	3	1		
C. baccatum var. *baccatum*	27	4	6		7	10					
C. baccatum var. *pendulum*	220	124	31	5	32	28					
C. pubescens	40	5	14		7	8	2	1	3		
**Wild**											
C. annuum var *glabriusculum*	29	11	15					3			
C. chacoense	29	9	5		1	9	4	1			
C. eximium	10	2	3		2	3					
C. praetermissum	9		5			3		1			
C. cardenasii	2		1			1					
C. tovarii	1					1					
C. galapagoense	3		2			1					
Undefined	266	258	5	2	1						
**TOTAL**	**10038**	**5351**	**1448**	**970**	**869**	**690**	**281**	**246**	**98**	**48**	**37**

WorldVeg = World Vegetable Center (WorldVeg, previously AVRDC - Asian Vegetable Research and Development Center Tainan, Taiwan)

IPK = Leibniz Institute of Plant Genetics and Crop Plant Research (Germany)

UPV-COMAV = Universitat Politècnica de València Germplasm Bank (Valencia, Spain)

INRAE = Centre de Ressources Biologiques Légumes de l'Unité de Génétique et Amélioration des Fruits et Légumes (GAFL, Montfavet, France)

CGN = Centre for Genetic Resources (Wageningen, The Netherlands)

ARO = Agricultural Research Organization Volcani Center (Israel)

BATEM = Batı Akdeniz Agricultural Research Institute (Turkey)

CREA = Research Centre for Vegetable and Ornamental Crops (, Italy)

MCVRI = Maritsa Vegetable Crops Research Institute (Bulgaria)

UNITO = University of Turin (Italy).

Table S2. F_{ST} weighted, genome wide estimation of expected (*He*) and observe (*Ho*) heterozygosity (values in percentage), inbreeding coefficient (*F*) and Tajima's D from non-overlapping 1-kb windows in the most represented *Capsicum* species and wild relatives.

Species	*C. frutescens*	*C. chinense*	*C. baccatum*	*C. pubescens*	*C. chacoense*	*C. annuum* var. *glabriusculum*	other Wilds*	*He*	*Ho*	*Ho* range	*F*	Tajima's D
C. annuum	0.787	0.803	0.873	0.805	0.790	0.245	0.806	12.041	0.417	10.177 - 0.000	0.965	-0.766
C. frutescens		0.566	0.781	0.650	0.607	0.567	0.615	12.202	0.928	8.852 - 0.004	0.923	0.284
C. chinense			0.808	0.695	0.632	0.607	0.642	12.194	0.900	10.057 - 0.006	0.925	-0.028
C. baccatum				0.785	0.737	0.800	0.658	12.017	0.664	8.716 - 0.008	0.945	-0.343
C. pubescens					0.679	0.693	0.536	11.971	0.255	0.440 - 0.135	0.979	0.553
C. chacoense						0.583	0.425	12.101	0.506	2.601 - 0.168	0.958	-0.302
C. annuum var. *glabriusculum*							0.558	11.951	0.502	4.059- 0.008	0.956	-0.002
Other wilds species*								12.087	0.413	0.985 - 0.070	0.966	0.259

* *C. eximium, C. galapagoense, G. tovarii, C. cardenasii, C. praetermissum*

Table S3. Average percentage of heterozygosity of the pepper species within each genebank. In brackets are indicated the minimum and maximum values encountered. The number of accessions for each species/genebank are reported in Table S1.

**Genebank**	***C. annuum***	***C. annuum var. glabriusculum***	***C. baccatum***	***C. baccatum var. baccatum***	***C. baccatum var. pendulum***	***C. chacoense***	***C. chinense***	***C. frutescens***	***C. pubescens***	***Undefined***	***Wild***
**INRAE**	0.13<br>*(0.00-4.11)*		0.46<br>*0.19 -0.88*	0.53<br>*(0.35-0.95)*	0.50<br>*(0.33-2.11)*	1.88<br>*(1.88-1.88)*	0.43<br>*(0.12-3.69)*	0.42<br>*(0.09-2.33)*	0.20<br>*(0.15-0.28)*	0.04<br>*(0.04 -0.04)*	0.42<br>*(0.40 -0.43)*
**IPK**	0.19<br>*(0.00-3.81)*	0.45<br>*0.04 -3.68*	0.24<br>*(0.12-0.35)*	0.41<br>*0.03 -0.94*	0.68<br>*0.18 -1.71*	0.22<br>*(0.17-0.28)*	0.34<br>*(0.08-1.06)*	0.38<br>*0.01 -2.95*	0.26<br>*(0.16-0.36)*	0.15<br>*(0.02 -0.25)*	0.38<br>*(0.24 -0.98)*
**UPV**	0.20<br>*(0.00-4.41)*		0.64<br>*(0.31-1.97)*		0.47<br>*(0.31-0.65)*		0.73<br>*(0.13-3.36)*	0.86<br>*0.01 -4.41*		0.38<br>*(0.36 -0.40)*	
**WorldVeg**	0.58<br>*(0.00-9.66)*	0.24<br>*0.01 -1.14*	0.91<br>*0.01 -6.39*	0.93<br>*(0.35-2.13)*	0.86<br>*0.03 -8.72*	0.52<br>*(0.17-1.17)*	1.16<br>*(0.08-10.06)*	1.23<br>*(0.09-9.91)*	0.31<br>*(0.14-0.44)*	2.46<br>*(0.00 -10.18)*	0.35<br>*(0.11 -0.59)*
**CGN**	0.26<br>*0.00 -6.16*			0.52<br>*(0.30-1.22)*	0.47<br>*0.24 -1.25*	0.35<br>*(0.17-0.75)*	0.49<br>*(0.06-5.95)*	0.37<br>*0.07 -3.16*	0.22<br>*(0.16-0.39)*		0.43<br>*(0.07-0.75)*
**ARO**	0.37<br>*0.00 -5.20*		0.52<br>*(0.33-0.85)*			0.91<br>*(0.28-2.60)*	1.85<br>*0.15 -6.88*	0.98<br>*(0.07-4.16)*	0.34<br>*(0.24-0.44)*		
**BATEM**	0.20<br>*(0.00-2.25)*										
**CREA**	0.93<br>*(0.00-6.10)*	1.73<br>*0.03 -4.06*	0.42<br>*(0.37-0.49)*			0.23<br>*(0.23-0.23)*	1.31<br>*(0.12-6.72)*	0.36<br>*(0.21-1.02)*	0.30<br>*(0.30-0.30)*		0.44<br>*(0.44 -0.44)*
**MVCRI**	0.17<br>*(0.00-1.77)*										
**UNITO**	0.22<br>*(0.00-2.08)*		0.29<br>*0.29 -0.29*				1.25<br>*(0.17-2.34)*		0.30<br>*(0.25-0.38)*		
Mean	0.42	0.50	0.71	0.56	0.72	0.51	0.90	0.93	0.26	2.40	0.40

Table S4. Discrimination according to best grouping according to ADMIXTURE analysis on 10,038 samples and 224 controls. Highlighted in bold, the main species and area of origin represented in each K groups. For instance, this table indicates that *C. annuum* accessions are majorly distributed in five main source populations (without considering the 224 samples of the CM334 controls).

K	N°	ANN	ANNg	FRU	CHI	BAC	BACb	BACp	PUB	CHA	EXI	PRA	CAR	TOV	GAL	*und*	AF#	AS	MA	SA	NA	EU	eEU	ME	OC	*na*
**K1**	0	224*															-	-	-	-	-	-	-	-	-	
**K2**	115	**95**	4	10	4	1										1			**100**	1	3	2				9
**K3**	794	74		**585**	30	1		2			1					101	62	**230**	**290**	96	20	15	3	1	5	72
**K4**	349	9	2	42	**288**	1		1							1	5	55	27	**72**	**112**	8	39	1			35
**K5**	315	18		32	**257**								1			7		3	**26**	**183**	18	15	10			60
**K6**	2849	**2819**	2	3	6	2										17	57	248	109	59	207	**1647**	215	144	5	158
**K7**	1882	**1860**		2	9			2								9	40	118	95	15	87	101	**1027**	297	1	101
**K8**	1835	**1690**	4	31	14			2								94	87	**1321**	44	25	70	99	21	59	5	104
**K9**	96	2	1	1	1		5		40	27	8	9	1	1				1	10	26	2	14	1			42
**K10**	410	15		2	3	**155**	**20**	**213**								2	3	10	**27**	**262**	24	31	6		1	46
**K11**	483	**443**	2	9	4	2										23	5	8	**373**	12	30	18	3			34
ADMIX	910	823	14	28	30	1	2			2	1				1	8	115	103	201	60	73	178	36	47	3	94
Total	10038	7848	29	745	646	163	27	220	40	29	10	9	2	1	2	267	424	2069	1347	851	542	2159	1323	548	20	755

ANN = *C.annuum* (with * are indicated the 224 control accessions); ANNg = *C. annuum var glabriusculum*; FRU = *C. frutescens*; CHI = *C. chinense*; BAC = *C. baccatum*; p = *var. pendulum*; b = *var. baccatum*; PUB = *C. pubescens* CHA = *C. chacoense*; EXI = *C. eximium*; PRA = *C. praetermissum*; CAR = *C. cardenasii*; TOV = *C. tovarii*; GAL = *C. galapagoense*; *und* = *undefined*

AF = AFRICA; AS = ASIA; MA= Mesoamerica; SA = South America; NA = North America; EU = Western and Central Europe; eEU = Eastern Europe; ME = Middle East; OC = Oceania; na = not available

Table S5. Discrimination according to best grouping following ADMIXTURE analysis on *C. annuum* samples. Highlighted, the main area of origin for the respective K groups.

K	N°	AF[#]	AS	MA	SA	NA	EU	eEU	ME	OC	*na*	Comments*
**K1**	1607	18	98	26	8	32	73	1004	275	1	72	**eEU**: Yugoslavia (373), Hungary (258,) Bulgaria (169), Albania (50); **ME**: Turkey (257)
**K2**	1151	58	885	22	16	33	42	8	47	3	37	**AS:** Thailand (229); India (213) Indonesia (118), Malasya (101)
**K3**	1153	9	183	35	21	162	480	120	50	3	90	**AS**: China (126); NA: USA (156); **EU**: Italy (186), Spain (145), France (61), Netherlands (50); **eEU:** Hungary (52)
**K4**	425	6	289	26	3	35	23	10	1		32	**AS:** Korea (155), China (66); Japan (53)
**K5**	439	5	7	344	5	30	13	3			32	**MA:** Mexico (107), Guatemala (69), El Salvador (68), Honduras (64)
**K6**	15	1	1	1	3		6	1			2	
**K7**	102	5	42	25	11	8		1			10	
**K8**	1379	49	21	39	32	35	1126	11	36		30	**EU:** Spain (877), Italy (185)
**K9**	95			83	1	3	2				6	
**ADMIX**	1482	148	191	285	54	119	259	133	132	6	155	**MA**: Mexico (154); **NA:** USA (118); **EU:** Italy (109), Spain (66); eEU: Hungary (60); **ME**: Turkey (103); **AF:** Ethiopia (92)
**Total**	7848	299	1717	886	154	457	2024	1291	541	13	466	

AF = AFRICA; AS = ASIA; MA= Mesoamerica; SA = South America; NA = North America; EU = Western and Central Europe; eEU = Eastern Europe; ME = Middle East; OC = Oceania; *na* = not available

* Countries holding > 50 accessions

Table S6. F_{ST} weighted, genome wide estimation of expected (*He*) and observe (*Ho*) heterozygosity (values in percentage), inbreeding coefficient (*F*) and Tajima's D from non-overlapping 1-kb windows in geographically defined germplasm groups of *C. annuum*.

Geographical regions	Asia South and SE*	Central Asia	Europe	Eastern Europe	Africa	Middle Est	Central America	North America	South America	N	*He*	*Ho*	*Ho* range	*F*	Tajima's D
**East Asia**	0.117	0.115	0.122	0.148	0.071	0.114	0.099	0.059	0.061	558	12.007	0.431	4.260 - 0.000	0.963	0.1992
**South/South East Asia**		0.201	0.236	0.242	0.098	0.188	0.111	0.143	0.120	1055	11.971	0.695	9.090 - 0.000	0.940	-0.6126
**Central Asia**			0.079	0.021	0.094	0.017	0.113	0.058	0.070	108	12.079	0.204	1.450 - 0.000	0.983	0.3052
**Western/Central Europe**				0.110	0.109	0.084	0.165	0.043	0.049	2030	12.051	0.248	6.100 - 0.000	0.979	-0.5552
**Eastern Europe**					0.133	0.033	0.173	0.093	0.117	1292	12.036	0.221	2.680 - 0.000	0.981	-0.8053
**Africa**						0.086	0.070	0.056	0.038	300	12.023	0.501	5.990 - 0.000	0.958	-0.4092
**Middle East**							0.124	0.063	0.073	541	12.054	0.291	3.630 - 0.000	0.976	0.0069
**Mesoamerica**								0.076	0.054	926	12.008	0.940	8.780 - 0.000	0.920	-0.3496
**North America**									0.017	458	12.051	0.335	4.560 - 0.000	0.972	-0.6225
**South America**										117	11.956	0.824	10.180 - 0.000	0.928	-0.8815

Table S7. k-mer-based GWAS analysis on 2059 *C. annuum* accessions reporting genomic regions highly significantly associated with genebank phenotyping data related to plant, flower and fruit traits.

Trait#	Chr	Position (Mbp)	Candidate gene	Coordinate	Distance*	pval
Annular Constriction	P1	306628047	hypothetical protein	306622565..306629396	0	2.30E-53
Fruit position	P12	212154217	Protein *ELF4*-LIKE 3	212145569..212152681	1.5 kb	1.95E-41
Fruit pungency	P2	149009267	hypothetical protein	149000773..149001185	8.1 kb	4.72E-14
Fruit pungency	P2	149009267	*PFP-BETA* (Pyrophosphate--fructose 6-phosphate 1-phosphotransferase subunit beta)	148965607:148973605	35 kb	4.72E-14
Fruit pungency	P2	150433374	*FSTH6* (ATP-dependent zinc metalloprotease FTSH (6,2) chloroplastic)	150431689..150434169	0	3.85E-11
Fruit pungency	P2	142582023	*DHNA-CoA* (1,4-dihydroxy-2-naphthoyl-CoA thioesterase 1)	142602782:142605457	20 kb	3.01E-10
Fruit shape	P10	67822247	*MSH5 (*DNA mismatch repair protein)	70464537:70542670	2642 Kb	1.66E-12
Fruit shape	P10	157397902	hypothetical protein	157390248:157390454	7.4 Kb	3.28E-12
Fruit shape	P11	155974461	hypothetical protein	155972621..155975491	0	9.84E-11
Fruit shape	P11	173506333	hypothetical protein	173493841..173510241	0	1.46E-10
Fruit shape	P11	162352950	putative glutamate carboxypeptidase 2	162329510..162355479	0	1.71E-10
Fruit shape	P11	142410707	DNA topoisomerase 3-alpha	142383641..142466172	0	4.40E-10
Number of pedicels per axil	P6	233943806	hypothetical protein	233940573..233947989	0	2.54E-29
Number of pedicels per axil	P6	233951395	UDP-D-apiose/UDP-D-xylose synthase 2; AP-2 complex subunit alpha-2	233972961..233976254	21.5 Kb	3.41E-23
Number of pedicels per axil	P6	234111001	SRSF protein kinase 2	234108662..234111657	0	8.19E-23
Number of pedicels per axil	P6	234083866	Protein SCO1-like protein, mitochondrial	234056380..234060539	23.3 Kb	1.17E-12
Pedicel position at anthesis	P2	164411993	*CONSTANS* protein	164420940..164423188	6105.8 Kb	2.13E-15
Pedicel position at anthesis	P12	212154217	hypothetical protein	212153938..212158694	0	1.65E-45
Pedicel position at anthesis	P12	212029248	Protein *ELF4*-LIKE 3	212053408..212053740	0	1.12E-27
Pedicel position at anthesis	P12	202407723	DEAD-box ATP-dependent RNA helicase 48	202388713..202416283	0	2.48E-12
Pedicel position at anthesis	P12	208007370	*FAB1B (*1-phosphatidylinositol-3-phosphate 5-kinase*)*	208001913..208013992	0	4.03E-11
Pedicel position at anthesis	P12	222550045	Inositol-tetrakisphosphate 1-kinase 3	222547255..222554435	0	6.66E-10

Traits are listed in alphabetical order. Description is reported in supplementary Table S9

* Distance from the nearest candidate gene. Distances = 0 means that the associated region fall within the gene

Table S8. Average trimmed and mapped reads for the studied *Capsicum* species, number of mapped regions using UMR (unmapped reads) q30 treshold and related percentage for each pepper species.

Species	Number	Average trimmed_reads	mapped_reads	umr_q30	umr_q30_%
C. annuum	8075	1007441.61	963089.16	548997.91	54.49
C. annuum var.*glabriusculum*	29	884201.28	830229.79	416488.28	47.10
C. baccatum	410	1016401.84	965488.77	458830.17	45.14
C. cardenasii	2	1705476.00	1651271.50	690631.50	40.49
C. chacoense	29	1011463.90	967754.21	473296.03	46.79
C. chinense	646	1009414.73	945219.15	479357.21	47.49
C. eximium	10	1087604.70	1048000.70	508653.70	46.77
C. frutescens	742	956420.21	894654.85	468114.33	48.94
C. galapagoense	3	722931.33	693214.33	327572.33	45.31
C. praetermissum	9	1120521.89	990904.89	493578.11	44.05
C. pubescens	40	1016319.50	956204.15	462972.35	45.55
C. tovarii	1	1042798.00	1019631.00	491844.00	47.17
C.sp (undefined)	266	913651.22	853858.09	459839.48	50.33

Table S9. Plant descriptors used for genebank phenotyping.

Trait	Scale values
**Plant traits**	
Plant growth habit	3:Prostrate, 5:Compact, 7:Erect
Plant height	1:Short (<50cm), 3:Intermediate (50-100cm), 5:Tall (>100cm), 7:Very tall (>200cm)
Nodal anthocyanin (whole plant)	0:Green, 1:Very light purple, 3:Light purple, 5:Purple, 7:Dark purple
**Flower Traits**	
Calyx margin shape	3:Smooth, 5:Intermediate, 7:Dentate
Corolla colour	1:White, 2:Light yellow, 3:Yellow, 4:Yellow-green, 5:White with purple base, 6:White with purple margin, 7:Purple, 8:Other (specify)
Corolla spot	0:Absent, 1:White, 2:Yellow, 3:Green-Yellow, 4:Green, 5. Other :Other
Flowering earliness	3:Early, 5:Medium, 7:Late
Number of pedicels per axil	1 to 7; fasciculate 9; mixture 10
Pedicel position at anthesis	3:Pendant, 5:Intermediate, 7:Erect
**Fruit traits**	
Annular constriction at junction of calyx and peduncle	0:Absent, 3:Not clear, 5:Clear
Fruit wall thickness	1:Very thin (<1mm), 3:Thin (1 < x < 2mm), 5:Medium (2 < x < 4mm), 7:Thick (4 < x < 6mm), 9:Very thick (> 6mm)
Fruit colour at immature stage	1:Green, 2:Yellow, 3:Orange, 4:Red, 5:Purple, 6:Brown, 7:Black, 8:Yellow-green, 9:Other (Specify), 10: white
Fruit colour at mature stage	1:Green, 11:Other (White), 2:Yellow, 3:Orange, 4:Orange-red, 4.5; light red; 5:Red, 5.5 dark red 6:Purple, 7:Brown, 8:Black, 9:Yellow-orange
Fruit cross-sectional corrugation (at 1/3 from pedicel end)	0:Smooth, 3:Slightly corrugated, 5:Intermediate, 7:Corrugated
Fruit position	3:Pendant, 5:Intermediate, 7:Erect
Fruit pungency	0:Not pungent (sweet), 1:Pungent
Fruit shape	1:Elongate, 2:Oblate, 3:Round, 4:Conical, 5:Campanulate, 6:Bell or blocky, 7:Pumpkin shaped, 8:Other (Specify)
Fruit shape at blossom end	3:Pointed, 5:Blunt, 7:Sunken, 9:Sunked and pointed
Fruit shape at peduncle attachment	1:Acute, 3:Obtuse, 5:Truncate, 7:Cordate, 9:Lobate
Fruit size*	1:Very small (<4g), 3:Small (4 < x < 10g), 5:Medium (10 < x < 40g), 7:Big (40 < x <150g), 9:Very big (>150g)
Maturity earliness	from 3 (early maturity) to 7 (late maturity)

*estimated according to weight ranges

Dataset S1 (separate file). Passport data of 10038 *Capsicum* accessions studied. ND = not determined.

Dataset S2 (separate file). Duplicated accessions and related clusters inferred by IBS.

Dataset S3 (separate file). Hierarchical clustering (z = 90) showing the percentage of each species in detected clusters. Highlighted those clusters comprising putative misclassified accessions.

Dataset S4 (separate file). Phenotypic observations in a core set of 2059 *C. annuum* accessions.
